# Supplementary material for: Single-crystal ZrCo nanoparticle for advanced hydrogen and H-isotope storage
Source: Nat Commun. 2023 Dec 2;14:7966. doi: 10.1038/s41467-023-43828-5 (PMC10693545; doi:10.1038/s41467-023-43828-5)
Supplement: Supplementary file 1 — Supplementary Information [file 41467_2023_43828_MOESM1_ESM.pdf]

Supplementary Information for

Single-crystal ZrCo nanoparticle for advanced hydrogen and H-isotope storage

Zhenyang Li, Shiyuan Liu, Yanhui Pu, Gang Huang, Yingbo Yuan, Ruiqi Zhu, Xufeng Li, Chunyan Chen, Gao Deng, Haihan Zou, Peng Yi, Ming Fang, Xin Sun, Junzhe He, He Cai, Jaxiang Shang, Xiaofang Liu*, Ronghai Yu*, Jianglan Shui*

Correspondence to: Xiaofang Liu, liuxf05@buaa.edu.cn; Ronghai Yu, rhyu@buaa.edu.cn; Jianglan Shui, shuijianglan@buaa.edu.cn

Contents:

Supplementary Methods

Supplementary Figures 1 to 52

Supplementary Tables 1 to 14

Supplementary References

Supplementary Methods

Van't Hoff equation.

According to the Pressure-Composition-Temperature (PCT) curves, the thermodynamic parameters, enthalpy (ΔH) and enthalpy (ΔS) were calculated using Van't Hoff[1] as follows Eq. (1):

$$\ln P_{\mathrm{eq}} = -\frac{\Delta H}{RT} + \frac{\Delta S}{R} \qquad (1)$$

where P_{eq} are the PCT equilibrium plateau pressure. The Arrhenius plots were nearly linear. The ΔH and ΔS values could be estimated from the slopes and intercepts of the lines.

Kissinger equation.

The dehydrogenation activation energy (E_{de}) was estimated using the Kissinger method (Eq. (2)) based on DSC data[2]:

$$\ln\left(\frac{\varphi}{T_P^2}\right) = -\frac{E_{de}}{RT_P} + \ln\left(\frac{AR}{E_{de}}\right) \qquad (2)$$

where T_p is absolute temperature at the maximum desorption rate, R is the gas constant, φ is the heating rate, and A is the frequency factor.

Bright- and dark-field TEM technology.

Bright- and dark-field TEM technology was used to study the distribution of disproportionation phase. First, objective aperture was used to select the electron beams passing through ZrCo particle, and bright-field TEM image and corresponding SAED pattern were obtained. After identifying the diffraction spots of ZrH_2 phase in the SAED pattern, the objective aperture was moved to only allow the diffraction beams

of ZrH_2 phase to pass through. The ZrH_2 phase presents bright spots due to its diffraction contrast in the dark-field TEM image, which can clearly show its distribution in the particle.

Computational methods.

Density functional theory (DFT) calculations were implemented in the Vienna ab initio simulation package (VASP) package[3]. The projector-augmented planewave (PAW) method was used to describe the electron-ion interactions[4]. The cutoff energy was set to 460 eV. Exchange and correlation effects were handled using the generalized gradient approximation (GGA) of the PBE scheme[5]. The Brillouin zone was set using the Monkhorst-Pack 5×5×5 k-points mesh. The results for the total energy and Hellmanne-Feynman forces were convergent within 10^{-6} eV and 10^{-2} eV/Å, respectively.

In order to incorporate the non-local, and long-ranged van der Waals (vdW) forces in the system of ZrCo and H_2, Girmme's DFT-D3 density functional is adopted in the calculation[6]. The surfaces with (3×3) supercell of ZrCo and $ZrCoH_3$ were separated by a vacuum slab of 15 Å. The bottom two layers were fixed, while other atoms in the top layers and the added hydrogen atoms were allowed to relax. For the grain boundary structure, the bottom two layers and the top two layers were fixed, while other atoms were allowed to relax. D and H atoms have identical electronic structure but different masses[7,8].

To investigate the kinetic characteristics and disproportionation difficulty at various positions in ZrCo and $ZrCoH_3$, H or D atom(s) was placed in ZrCo and

$ZrCoH_3$ at different locations and the corresponding energy was calculated as Eqs. (3) and (4). The formation energy E_f of ZrCo (110) with H or D atom was calculated as:

$$E_f = E_{ZrCo+H/D} - E_{ZrCo} - \frac{1}{2} E_{H_2/D_2} \tag{3}$$

where E_{ZrCo} refers to the total energy of ZrCo (110), $E_{ZrCo+H/D}$ denotes the total energy of ZrCo (110) with one H or D atom absorbed on the surface or inserted in the subsurface, and E_{H_2/D_2} is the total energy of an isolated H_2 or D_2 molecule. The insertion energy E_f of H or D at surface and boundary region of $ZrCoH_3$ was calculated as:

$$E_f = E_{ZrCoH_3+H/D} - E_{ZrCoH_3} - \frac{n}{2} E_{H_2/D_2} \tag{4}$$

where E_{ZrCoH_3} refers to the total energy of the surface or boundary of $ZrCoH_3$, $E_{ZrCoH_3+H/D}$ denotes the total energy of the surface or boundary of $ZrCoH_3$ with H or D atom(s) absorbed/inserted at surface or boundary region. n is the number of the added H or D atom, and E_{H_2/D_2} is the total energy of an isolated H_2 or D_2 molecule.

Electromagnetic interference shielding performance.

When microwaves are incident on a material, the incident waves can be reflected, scattered and absorbed by the material, and the remaining waves are transmitted through the material (as shown in Fig. 5a). If the wavelength of microwaves is much larger than the average size of the rough features on the surface of lossy materials, scattering can be ignored. Hence, the power of the

incident wave (P_I) can be divided into three parts: the power of the reflected wave (P_R), the power of the absorbed wave (P_A), and the power of the transmitted wave (P_T)[9]. The protection of a material against the incident microwaves is defined as electromagnetic interference shielding effectiveness (EMI SE), which is expressed as the ratio of incident power to transmitted power on a logarithmic scale, as shown in Eq. (5)[10].

$$SE(\text{dB}) = 10\log(\frac{P_I}{P_T}) = 10\log(\frac{1}{T}) \qquad (5)$$

where T is the transmission coefficient. For a two-port vector network analyzer, S-parameters S_{11} (S_{22}) and S_{12} (S_{21}) were measured, which represent the reflection coefficient (R) and transmission coefficient given as follows:

$$T = |S_{21}|^2 = |S_{12}|^2 \qquad (6)$$

$$R = |S_{11}|^2 = |S_{22}|^2 \qquad (7)$$

Therefore, the SE can be derived from the measured scattering parameters S_{21}:

$$SE = 10\log\left(\frac{1}{|S_{21}|^2}\right) \qquad (8)$$

The logarithmic relationship with dehydrogenation temperature and particle size.

For the $ZrCoH_3$ dehydrogenation system ($ZrCoH_3 \rightarrow ZrCo + H_2$), the molar Gibbs free energy change ($\Delta_r G_m$) is the sum of bulk phase $\Delta_r G^b_m$ (the superscript b denotes bulk quantities) and surface phase $\Delta_r G^s_m$ (the superscript s denotes surface quantities)[11,12],

$$\Delta_r G_m = \Delta_r G_m^b + \Delta_r G_m^s \,. \qquad (9)$$

In Eq. (9),

$$\Delta_r G_m^b = \Delta_r H_m^b - T\Delta_r S_m^b \,, \qquad (10)$$

where $\Delta_r H^b_m$ and $\Delta_r S^b_m$ are the molar reaction enthalpy and molar reaction entropy corresponding to the bulk phase in reaction systems, respectively.

Assuming that the particles of the dispersion system are spherical particles, if the system consists of *n* components with *n*=1, 2, …, N, $\Delta_r G^s_m$ is expressed as[13]:

$$\Delta_r G_m^s = \sum_B \frac{2\nu_B \sigma_B M_B}{\rho_B r_B} = \sum_B \frac{2\nu_B \sigma_B V_{m_B}}{r_B}, \tag{11}$$

where ν_B, σ_B, M_B, V_{m_B}, ρ_B and r_B denote the stoichiometric number, surface tension, molar mass, molar volume, density and radius of component *B*, respectively. After substituting Eqs. (10) and (11) into Eq. (9), it becomes

$$\Delta_r G_m = \Delta_r H_m^b - T\Delta_r S_m^b + \sum_B \frac{2\nu_B \sigma_B V_{m_B}}{r_B}. \tag{12}$$

For this system, the hydride can decompose only when $\Delta_r G_m \leq 0$ under the conditions of constant temperature and pressure. Thus, the dehydrogenation temperature appears when $\Delta_r G_m = 0$, and Eq. (13) is expressed as:

$$\Delta_r H_m^b - T\Delta_r S_m^b + \sum_B \frac{2\nu_B \sigma_B V_{m_B}}{r_B} = 0. \tag{13}$$

Since the products (ZrCo, H_2) formed at the beginning of the dehydrogenation reaction can be neglected, only the item of the reactant ($ZrCoH_3$) is left in the sum. Therefore, Eq. (13) can be simplified as:

$$\Delta_r H_m^b - T\Delta_r S_m^b + \frac{2\nu\sigma V_m}{r} = 0. \tag{14}$$

$\Delta_r H^b_m$, $\Delta_r S^b_m$ and V_m are functions of *T*. σ and V_m are the surface tension and molar volume of $ZrCoH_3$. If *T* is regarded as an intermediate variable, the relationship between dehydrogenation temperature and particle radius can be derived from the partial derivative of Eq. (14) against *r*, as follows:

$$\left(\frac{\partial \Delta_r H_m^b}{\partial T}\right)_p \left(\frac{\partial T}{\partial r}\right)_p - \Delta_r S_m^b \left(\frac{\partial T}{\partial r}\right)_p - T\left(\frac{\partial \Delta_r S_m^b}{\partial T}\right)_p \left(\frac{\partial T}{\partial r}\right)_p + 2\nu\left[\frac{\partial}{\partial r}\left(\frac{\sigma V_m}{r}\right)\right]_p = 0. \tag{15}$$

Here,

$$\left(\frac{\partial \Delta_r H_m^b}{\partial T}\right)_p = \Delta C_{p,m}\,, \tag{16}$$

$$\left(\frac{\partial \Delta_r S_m^b}{\partial T}\right)_p = \frac{\Delta C_{p,m}}{T}\,, \tag{17}$$

and

$$\left[\frac{\partial}{\partial r}\left(\frac{\sigma V_M}{r}\right)\right]_p = \frac{r[\frac{\partial(\sigma V_M)}{\partial r}]_p - \sigma V_M \cdot \left(\frac{\partial r}{\partial r}\right)_p}{r^2}$$

$$= \frac{r\left[\sigma \cdot \left(\frac{\partial V_M}{\partial r}\right)_p + V_M \cdot \left(\frac{\partial \sigma}{\partial r}\right)_p\right] - \sigma V_M}{r^2}\,, \tag{18}$$

$$\left(\frac{\partial V_M}{\partial r}\right)_p = \left(\frac{\partial V_M}{\partial T}\right)_p \left(\frac{\partial T}{\partial r}\right)_p\,. \tag{19}$$

$\Delta C_{p,m}$ is the difference between the molar heat capacity of the product and reactant under a constant pressure.

Since σ is a function of T and r, i.e. $\sigma(T, r)$,

$$\left(\frac{\partial \sigma}{\partial r}\right)_p = \left(\frac{\partial \sigma}{\partial T}\right)_p \left(\frac{\partial T}{\partial r}\right)_p + \left(\frac{\partial \sigma}{\partial r}\right)_p \left(\frac{\partial r}{\partial r}\right)_p\,. \tag{20}$$

Therefore, Eqs. (18)-(20) can be combined into

$$\left[\frac{\partial}{\partial r}\left(\frac{\sigma V_m}{r}\right)\right]_p = \frac{r\left[\sigma\left(\frac{\partial V_m}{\partial T}\right)_p \left(\frac{\partial T}{\partial r}\right)_p + V_m\left(\frac{\partial \sigma}{\partial T}\right)_p \left(\frac{\partial T}{\partial r}\right)_p + V_m\left(\frac{\partial \sigma}{\partial r}\right)_p\right] - \sigma V_m}{r^2}\,. \tag{21}$$

Finally, Eqs. (15)-(17), (21) can be combined into

$$\left(\frac{\partial T}{\partial r}\right)_p = \frac{2\nu V_m\left[\sigma - r\left(\frac{\partial \sigma}{\partial r}\right)_p\right]}{rW - r^2 \Delta_r S_m^b}\,, \tag{22}$$

where

$$W = 2\nu\left[\left(\frac{\partial \sigma}{\partial T}\right)_p V_m + \left(\frac{\partial V_m}{\partial T}\right)_p \sigma\right]. \tag{23}$$

According to the Tolman equation $\sigma = \sigma_\infty / (1 + 2\delta / r)$ (σ_∞ is surface tension of the corresponding bulk substance, δ is the Tolman parameter which is on the order of 10^{-10} m), the size effect of surface tension becomes notable only when the size is less than 10 nm[14,15]. Thus, the effect of radius on surface tension can be neglected when the particle size is larger than 10 nm (i.e. $\frac{\partial\sigma}{\partial r} \approx 0$). Eq. (22) can thus be written as

$$\left(\frac{\partial T}{\partial r}\right)_p = \frac{2\nu\sigma V_m}{rW - r^2\Delta_r S_m^b}, \qquad (24)$$

Eq. (24) is the differential relationship between dehydrogenation temperature and particle radius, which can be solved by numerical differentiation. Integrating Eq. (25) with particle radius from ∞ to R and temperature from dehydrogenation temperature of bulk particle (T^b) to dehydrogenation temperature of nanoparticle (T), the following equation is obtained:

$$T - T^b = -\frac{2\nu\sigma V_m}{W}\ln\left(1 - \frac{W}{R\Delta_r S_m^b}\right). \qquad (25)$$

In Eq. (25), the order of magnitude of $\left(\frac{\partial\sigma}{\partial T}\right)_p$ is 10^{-4} [16], V_M is $\sim 3\times10^{-5}$ m^3 mol^{-1}[11], the order of magnitude of $\left(\frac{\partial V_M}{\partial T}\right)_p$ is $\sim 10^{-10}$ [11], σ is ~2 N m^{-1} [16], $\Delta_r S^b_m$ is ~200 J mol^{-1} K^{-1} (Supplementary Table 10) for nanoscale ZrCo spherical particles. Therefore, the order of magnitude for W value is $\sim 10^{-9}$. For the ZrCo spherical particles, R is in the range of 67 nm~621 nm. Hence, the $\frac{W}{R\Delta_r S_m^b}$ value can be estimated as ~0. According to Taylor formula [i.e. $\ln(1-x) = -x + o(x)$], we can simplify Eq.(25) as:

$$T - T^b = \frac{2\nu\sigma V_m}{R\Delta_r S_m^b}. \qquad (26)$$

It is worth noting that Eq. (26) is obtained based on a spherical particle model. Therefore, in our experiment, this equation is applicable to chemically-synthesized ZrCo particles below 1 μm.

If the particles are non-spherical, we should reconsidered the effect of particle shape on $\Delta_r G^s_m$[13, 17].

$$\Delta_r G_m^s = \sum_B \frac{\Omega \nu_B \sigma_B M_B}{\rho_B r_B} = \sum_B \frac{\Omega \nu_B \sigma_B V_{m_B}}{r_B}, \quad (27)$$

where Ω is the shape factor. Therefore, Eq. (26) should be written as

$$T - T^b = \frac{\Omega \nu \sigma V_m}{R \Delta_r S_m^b}. \quad (28)$$

In our experiment, Eq. (28) is applicable to the ZrCo particles above 1μm. Comparing Eqs. (26) and (28), we can conclude that there is an approximate linear relationship between the decomposition temperature and reciprocal of particle radius, but the slope of the T vs. $1/R$ curve is related to the shape of the particles.

Supplementary Figures and Tables

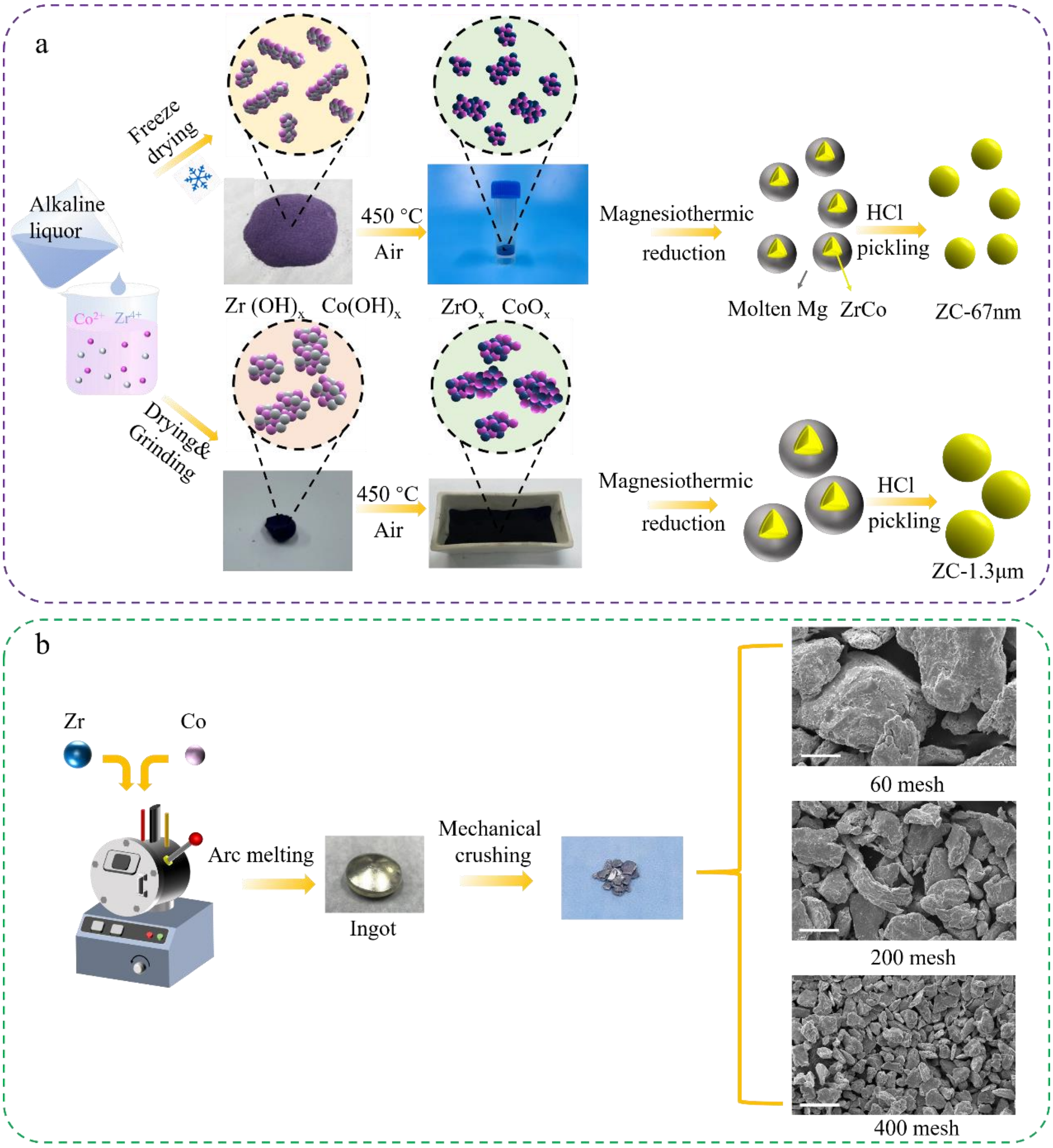

Supplementary Figure 1. Schematic illustration of the preparation of ZrCo alloys: (**a**) chem-ZrCo, and (**b**) smelting-ZrCo, the scale bars are 100 μm.

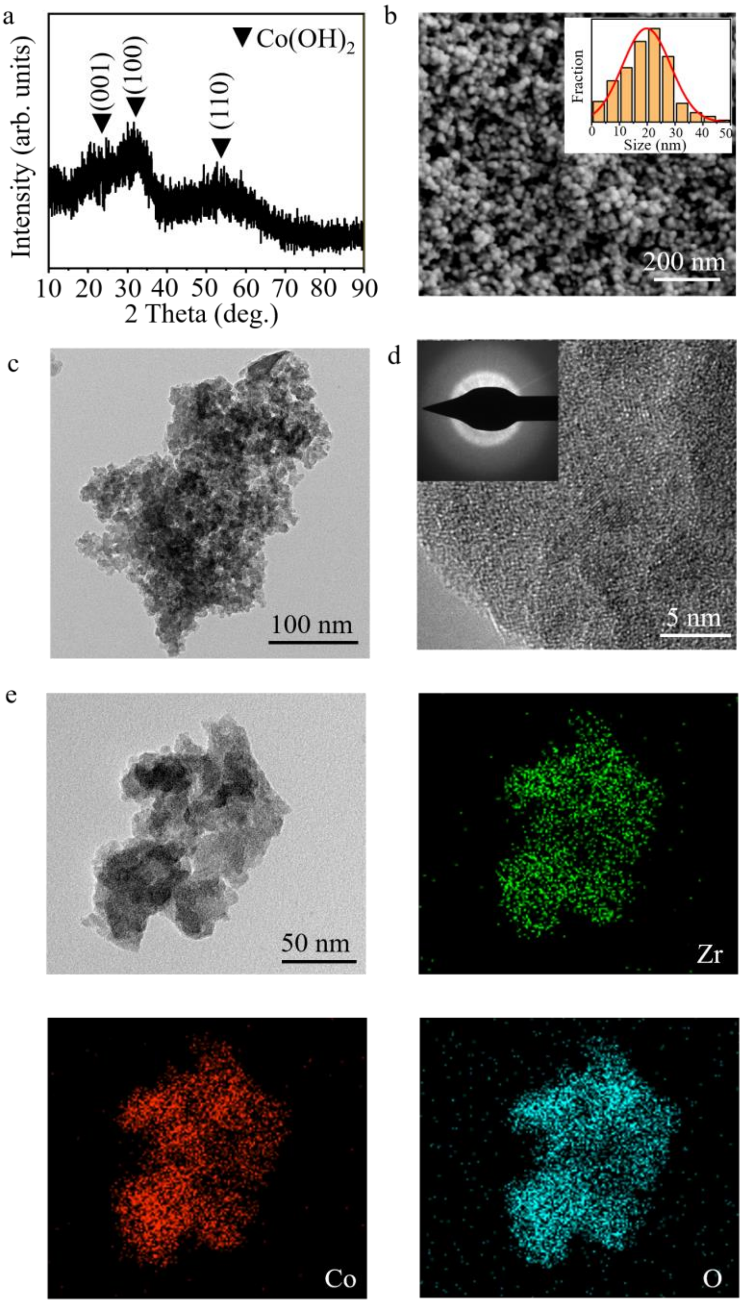

Supplementary Figure 2. Characterizations of zirconium-cobalt hydroxides. (**a**) XRD pattern; (**b**) SEM image; (**c**) TEM image; (**d**) HRTEM and SAED images; (**e**) elemental mappings.

XRD pattern, HRTEM and SAED images show that the zirconium-cobalt hydroxides are almost amorphous. SEM image reveals that the average particle size of hydroxide is 19.7 nm. Element mapping results indicate that zirconium and cobalt elements are uniformly mixed together.

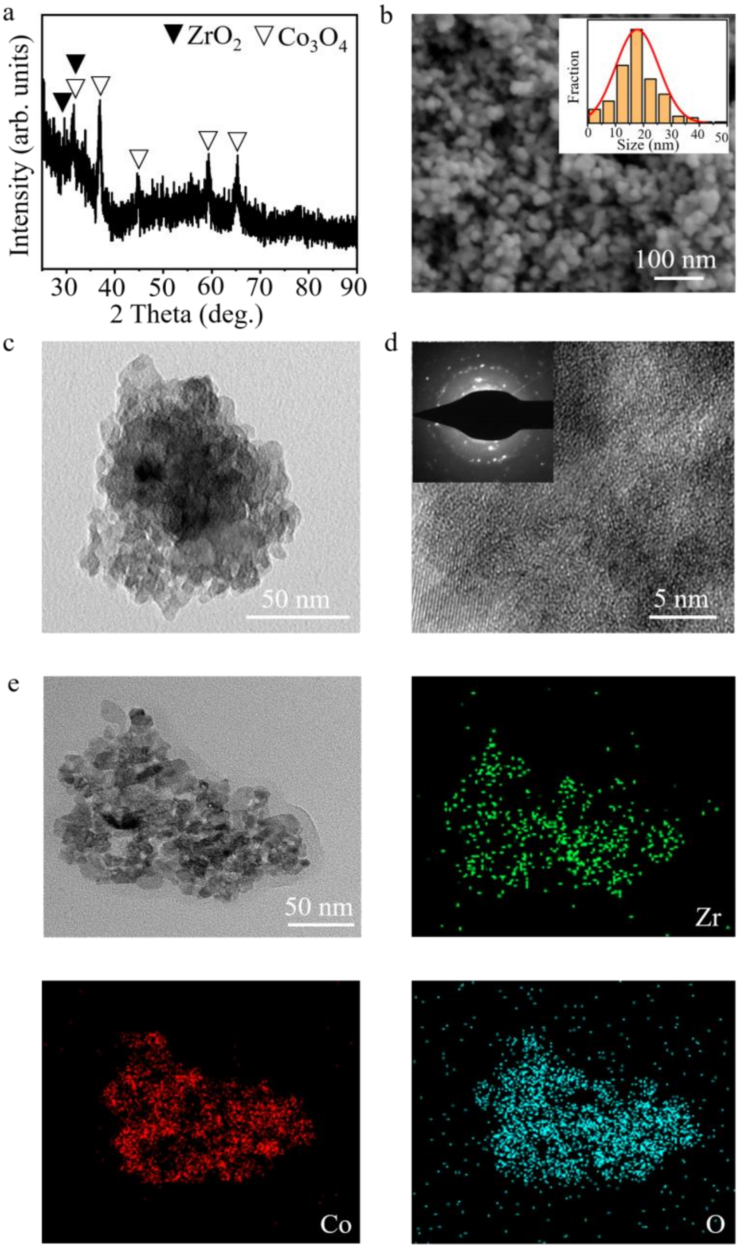

Supplementary Figure 3. Characterizations of zirconium-cobalt oxides. (**a**) XRD pattern; (**b**) SEM image; (**c**) TEM image; (**d**) HRTEM and SAED images; (**e**) elemental mappings.

After calcination in air, the zirconium-cobalt hydroxides were converted to oxides. The XRD pattern shows weak diffraction peaks of ZrO_2 and Co_3O_4 phases, indicating the poor crystallinity. SEM image reveals that the particle size of oxide is about 18 nm. TEM and SAED images further show the poor crystallinity of the oxides. Element mapping results indicate that zirconium and cobalt elements are uniformly mixed together.

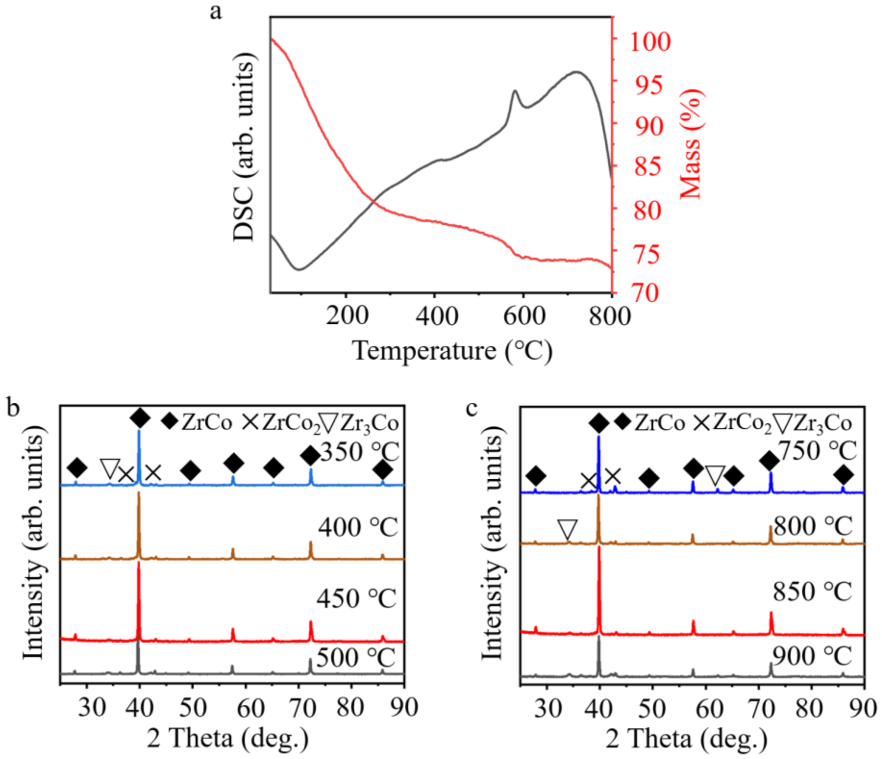

Supplementary Figure 4. Optimization of the heat treatment temperatures of ZC-67nm. (**a**) TG and DSC curves of zirconium-cobalt hydroxides; (**b**) XRD patterns of the final samples subjected to different oxidation temperatures (The magnesiothermic reduction temperature was 850 °C); (**c**) XRD patterns of the final samples obtained at different magnesiothermic reduction temperatures (The oxide precursors were obtained at 450 °C).

DSC curve shows an endothermic peak at ~100 °C, which can be attributed to the dehydration of zirconium-cobalt hydroxides. As the temperature rose, the sample was gradually oxidized and released heat, producing an exothermic peak between 550 and 600 °C. According to the weight loss of the precursor at different temperatures, we selected the oxidation temperatures of 350, 400, 450 and 500 °C. According to the XRD patterns of the final samples subjected to different oxidation temperatures (Supplementary Fig. 4b), the optimal oxidation temperature is deduced to be 450 °C, because the corresponding final product has better crystallinity and higher purity. Supplementary Fig. S4c shows that the final product reduced at 850 °C has higher crystallinity and purity. Thus the optimal magnesiothermic reduction temperature is determined to be 850 °C.

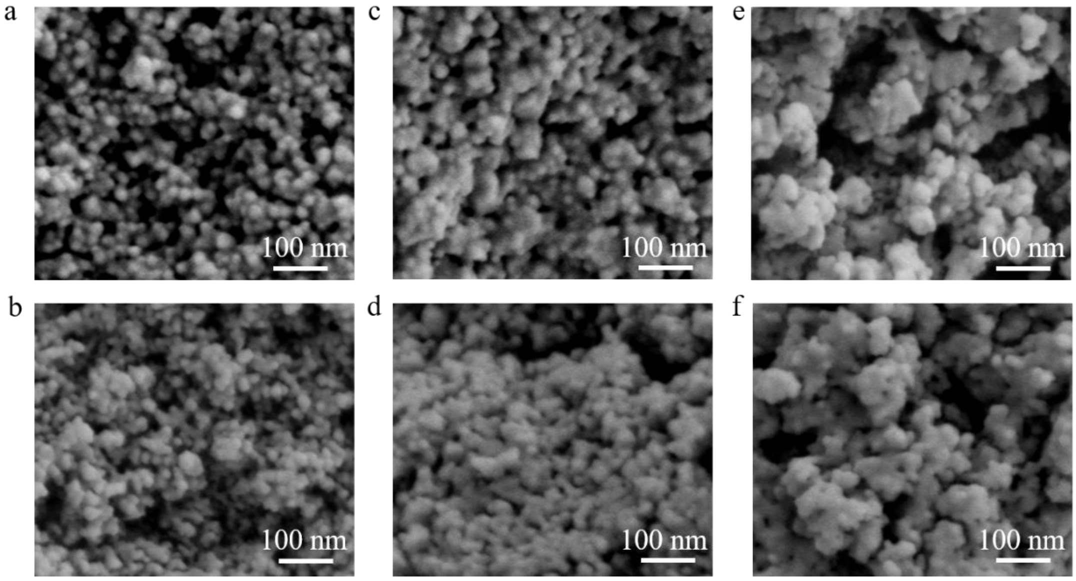

Supplementary Figure 5. SEM images of zirconium-cobalt hydroxides (up row) and oxides (low row) for (**a, b**) ZC-67nm; (**c, d)** ZC-336nm; (**e, f**) ZC-621nm.

The hydroxides shown in Supplementary Fig. 5 were subjected to freeze-drying to avoid severe agglomeration of nanoparticles. In addition, the size of hydroxide particles can be well adjusted by controlling the concentration of metal salt solution. As the salt concentration increases, the size of the hydroxide particles increases and the size of the calcined oxide particles increases accordingly.

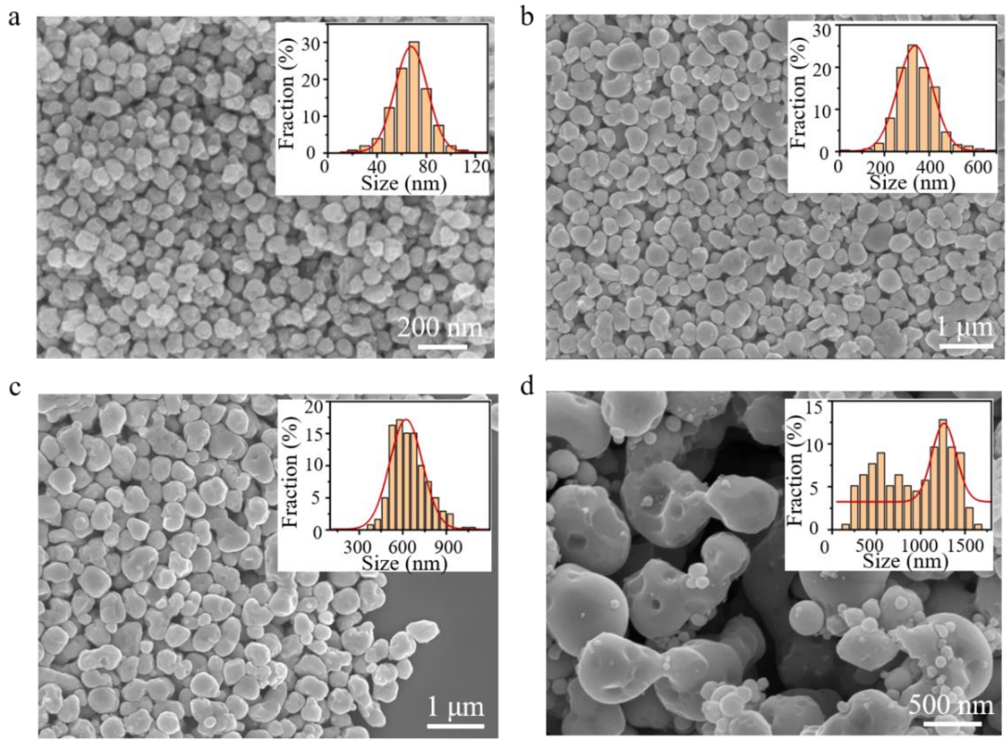

Supplementary Figure 6. SEM images of ZrCo alloys prepared by wet chemical method. (**a**) ZC-67nm; (**b**) ZC-336nm; (**c**) ZC-621nm and (**d**) ZC-1.3μm. Inset is the distribution of particle size.

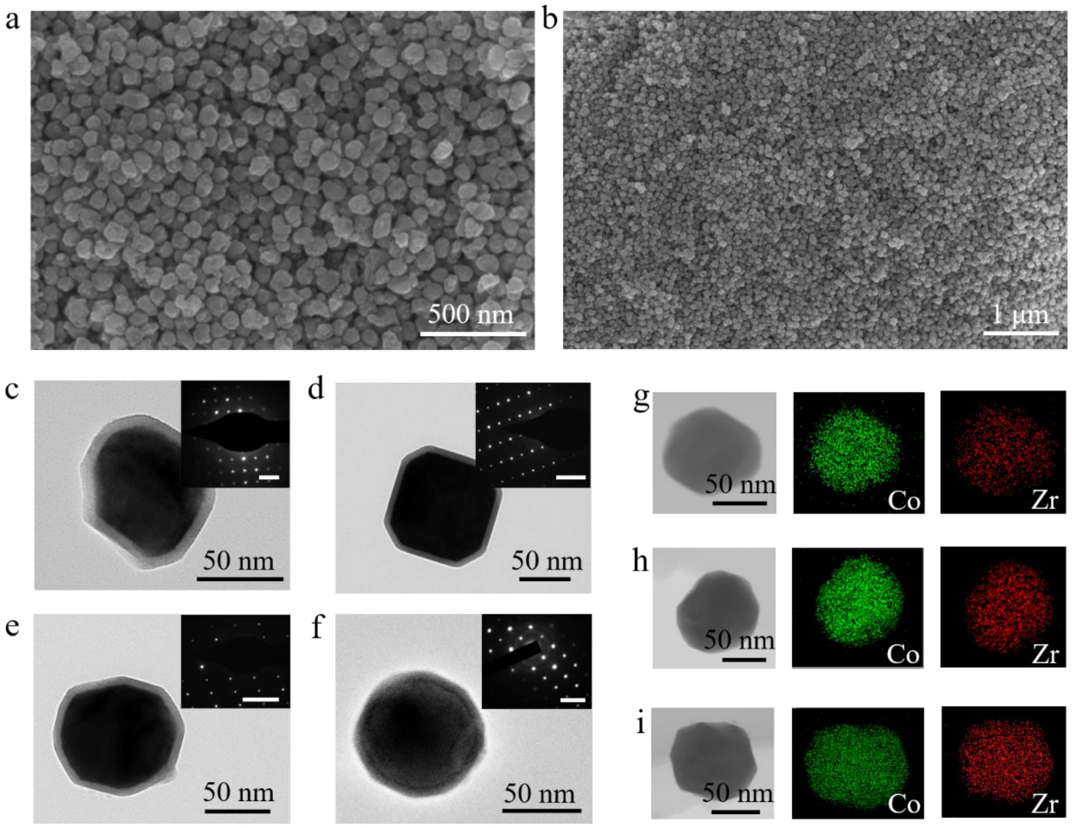

Supplementary Figure 7. (**a, b**) SEM images; (**c, d, e, f**) TEM and SAED images, the scale bars of SAED images are 5 1/nm; (**g, h, i**) elemental mappings of ZC-67nm.

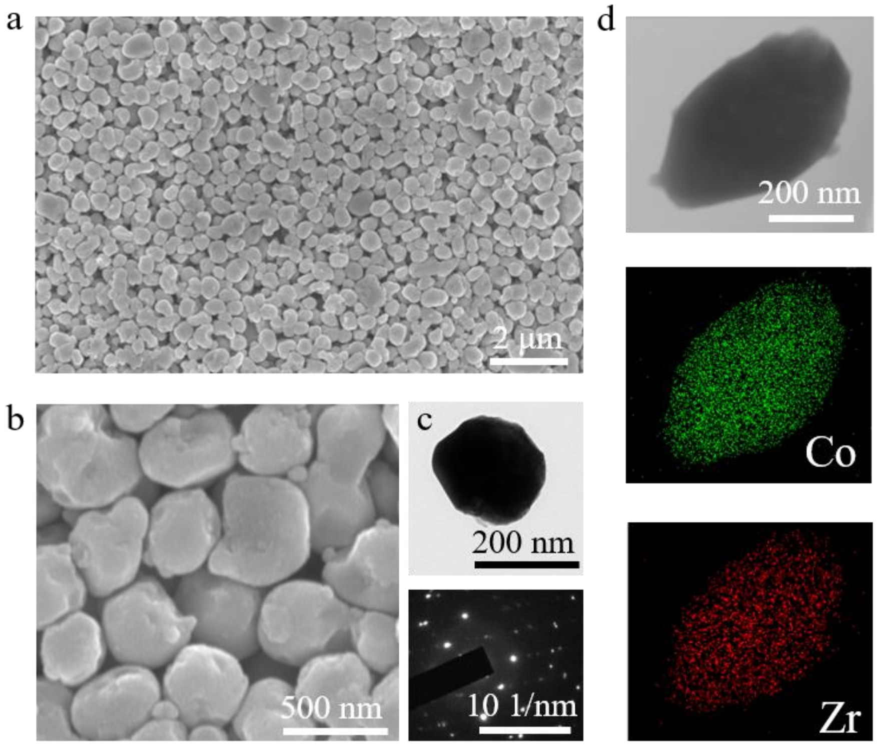

Supplementary Figure 8. (**a, b**) SEM images; (**c**) TEM and SAED images; (**d**) elemental mappings of ZC-336 nm.

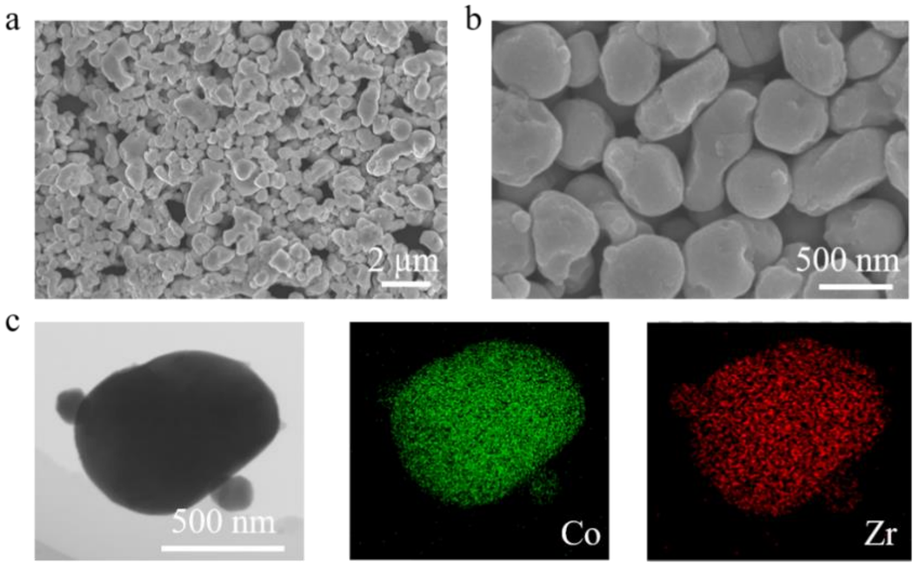

Supplementary Figure 9. (**a, b**) SEM images; (**c**) elemental mappings of ZC-621nm.

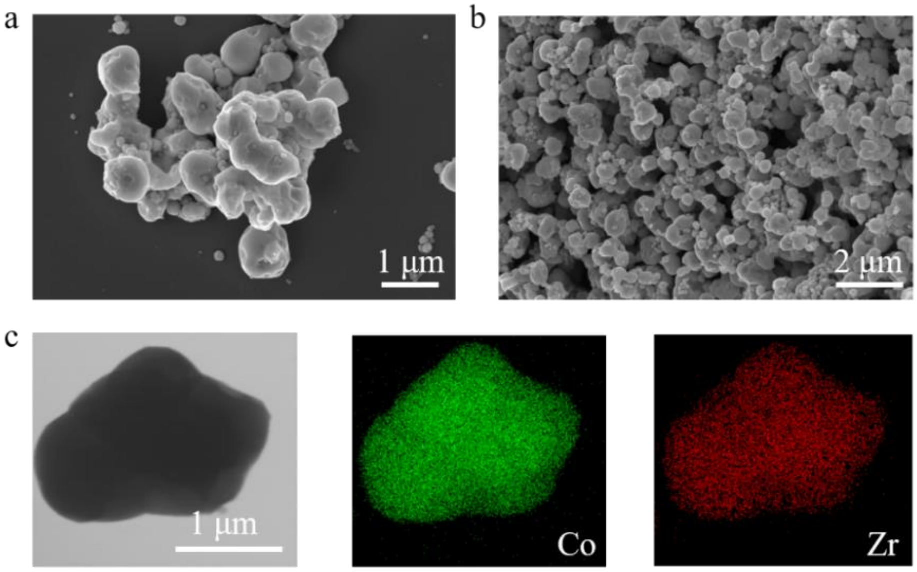

Supplementary Figure 10. (**a, b**) SEM images; (**c**) elemental mappings of ZC-1.3μm.

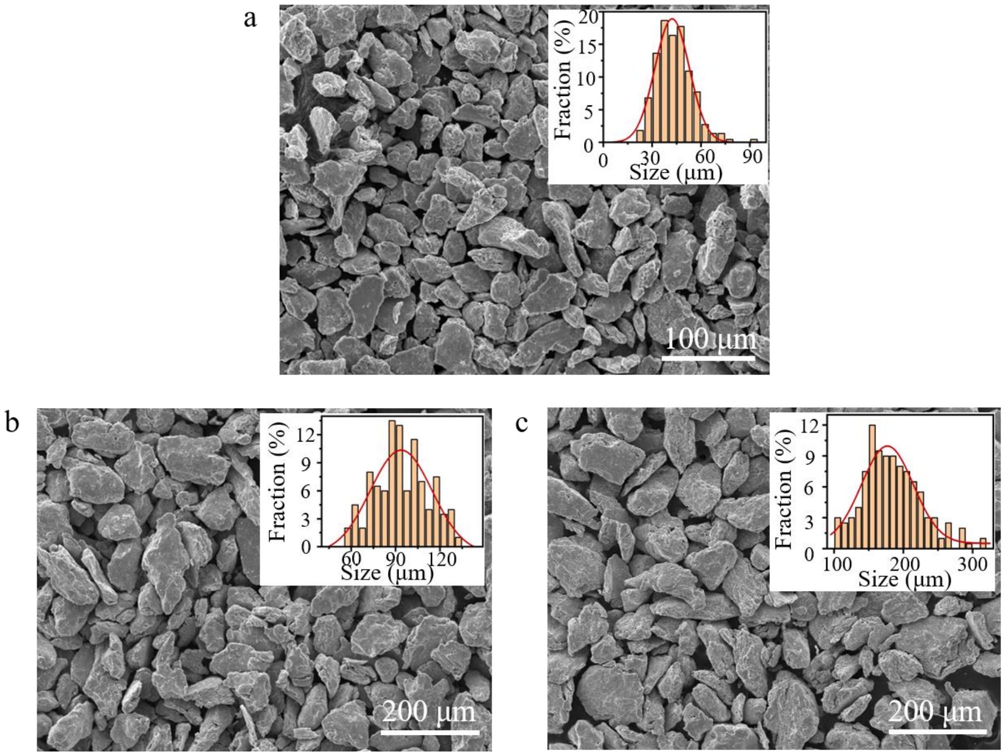

Supplementary Figure 11. SEM images of ZrCo alloys prepared by smelting method. (**a**) ZC-42μm; (**b**) ZC-94μm; (**c**) ZC-177μm. Inset is the distribution of particle size.

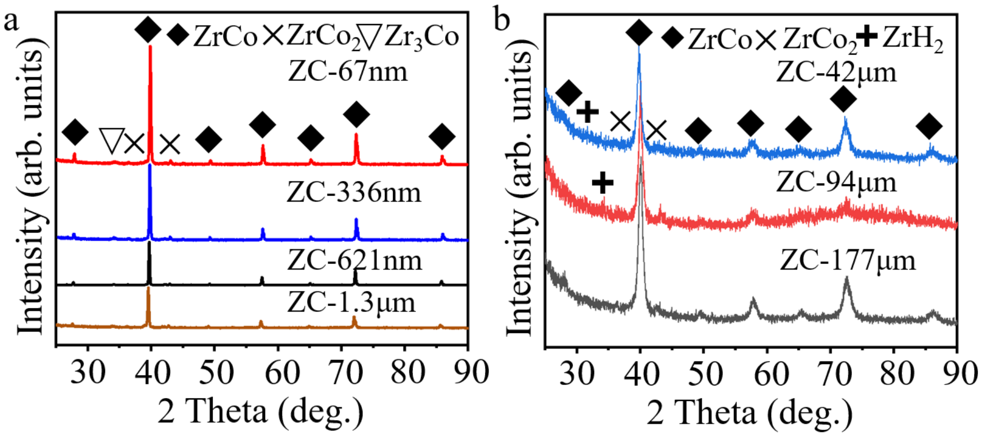

Supplementary Figure 12. XRD patterns of (**a**) chem-ZrCo and (**b**) smelting-ZrCo.

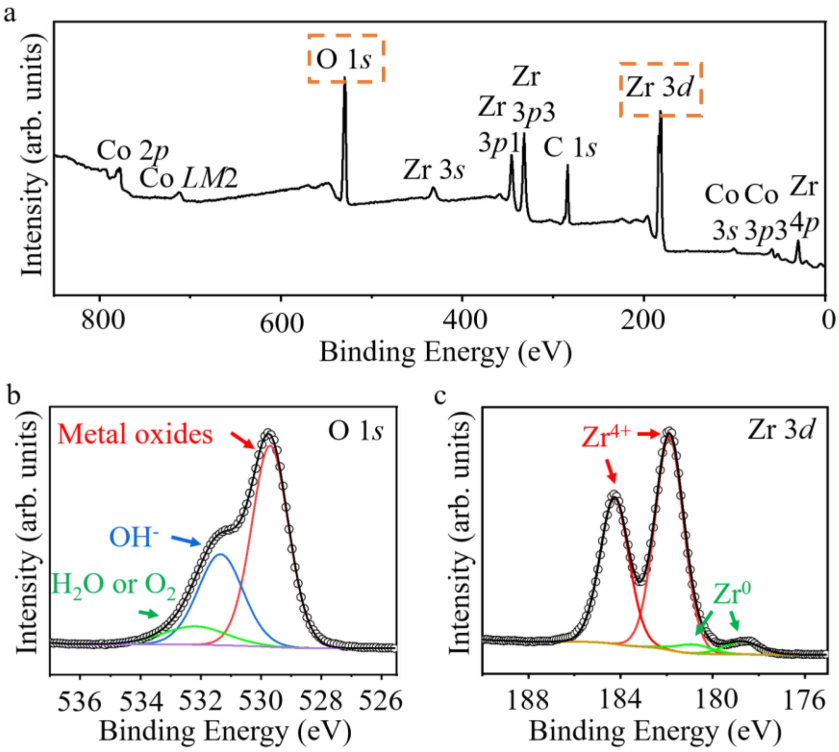

Supplementary Figure 13. XPS spectra of ZC-67nm. (**a**) Full spectrum; (**b**) O 1*s* spectrum; (**c**) Zr 3*d* spectrum.

XPS spectra indicate that the concentration of Zr is much higher than that of Co on the surface of ZC-67nm. The high-resolution O 1s spectrum contains three peaks at 529.7, 531.4 and 532.2 eV, deriving from metal oxides, OH^-, and adsorbed O_2/H_2O, respectively. The high-resolution Zr 3*d* spectrum reveals that Zr is mainly in the +4 oxidation state on the surface, corresponding to ZrO_2. The weak peaks of Zr^0 originate from the internal ZrCo alloy. This oxide surface originates from the air oxidation.

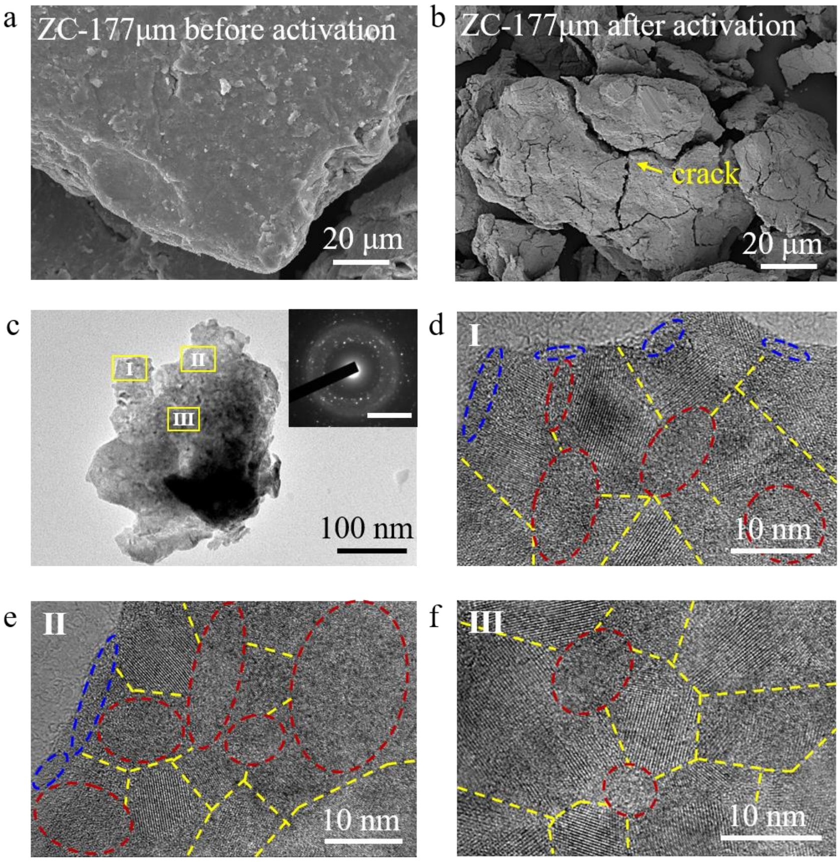

Supplementary Figure 14. SEM images of ZC-177μm (**a**) before activation, (**b**) after activation; (**c**) TEM and SAED images of ZC-177μm after activation; (**d-f**) the HRTEM of the areas of I, II and III in (c). Red circles indicate inhomogeneities (amorphous phases); blue circles indicate phase boundaries (between metal and oxides); yellow circles indicate grain boundaries.

The activated ZC-177μm particles are polycrystalline, and contain cracks, phase boundaries, grain boundaries and amorphous phase.

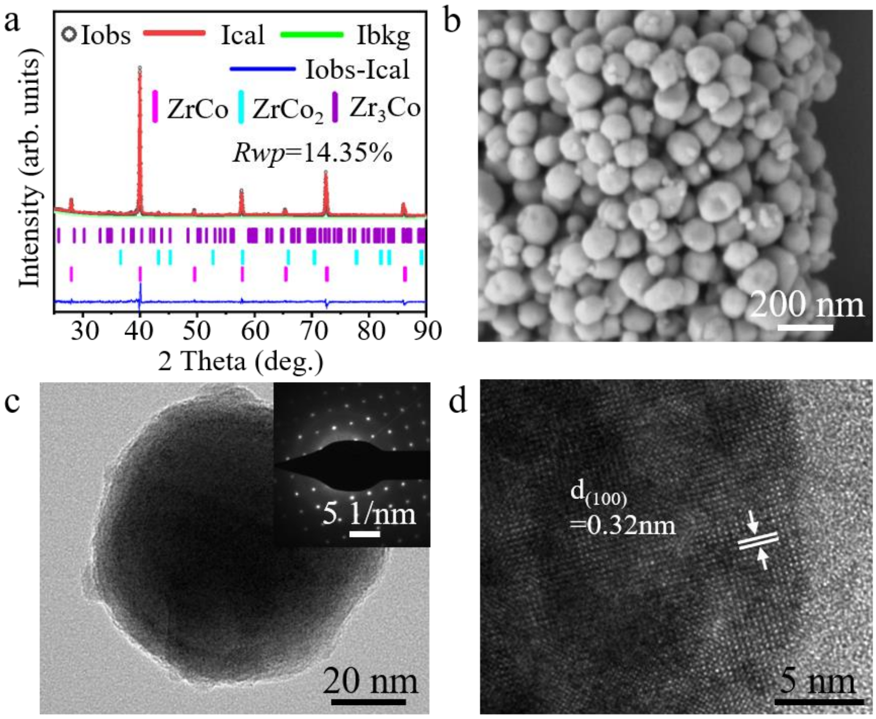

Supplementary Figure 15 (**a**) XRD patterns and Rietveld refinement result (**b**) SEM, (**c**) TEM, SAED and (**d**) HRTEM images of ZC-67nm after the initial hydrogenation/dehydrogenation cycle.

Supplementary Table 1. Rietveld refinement results of XRD patterns of ZC-67nm after the initial hydrogenation/dehydrogenation

**Sample**	**Lattice constant of ZrCo phase (Å)**	**Cell volume of ZrCo phase (Å^3)**	**Phase abundance (wt.%)**		
			**ZrCo**	**$ZrCo_2$**	**Zr_3Co**
ZC-67nm after 1 cycle.	3.198	32.703	95.2	3.7	1.1

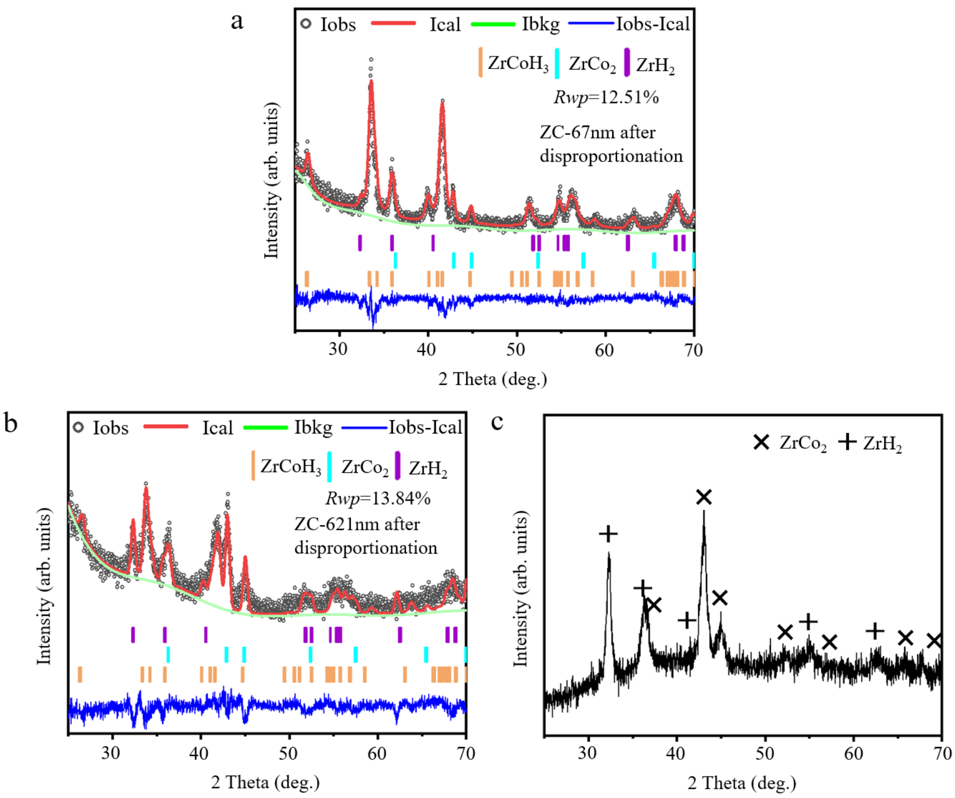

Supplementary Figure 16. Rietveld refinements of XRD patterns of (**a**) ZC-67nm after disproportionation, (**b**) ZC-621nm after disproportionation. (**c**) XRD pattern of ZC-177μm after disproportionation.

Supplementary Table 2. Rietveld refinement results of XRD patterns of ZC-67nm and ZC-621nm after disproportionation.

**Sample**	**Phase abundance (wt.%)**		
	**$ZrCoH_3$**	**$ZrCo_2$**	**ZrH_2**
ZC-67nm	80.4	10.9	8.7
ZC-621nm	44.4	30.8	24.8

Supplementary Table 3. A summary of crystallite size (calculated according to Rietveld refinement) and particle size of ZrCo alloys after activation.

**Sample**	**Crystallite size (nm)**	**Particle size**
ZC-67nm	50.1	67 nm
ZC-336nm	47.6	336 nm
ZC-621nm	42.5	621 nm
ZC-1.3μm	39.8	1.3 μm
ZC-42μm	30.9	42 μm
ZC-94μm	25.1	94 μm
ZC-177μm	29.0	177 μm

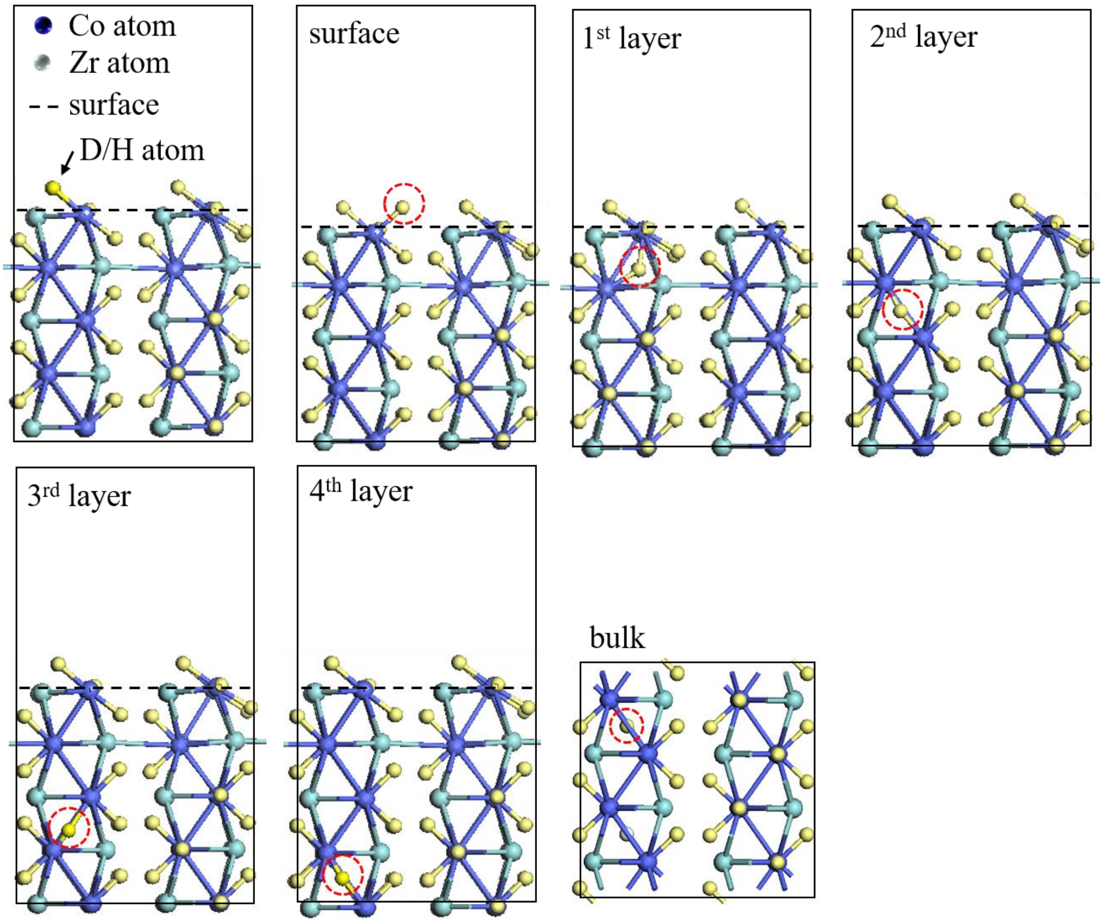

Supplementary Figure 17. Side views of $ZrCoH_3$ (002) with an additional H/D inserted. The red circle indicates the inserted H/D atom.

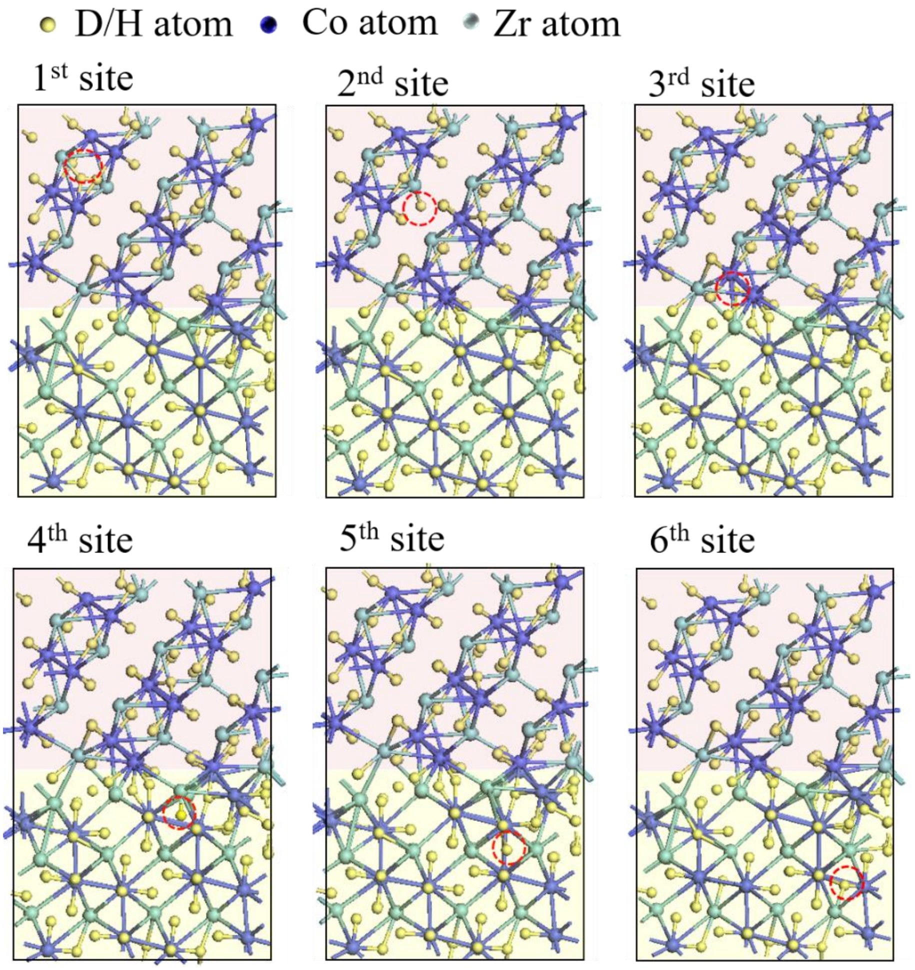

Supplementary Figure 18. Side views of the boundary of $ZrCoH_3$ with an additional H/D inserted. The red circle indicates the inserted H/D atom. Two different background colors indicate different orientations.

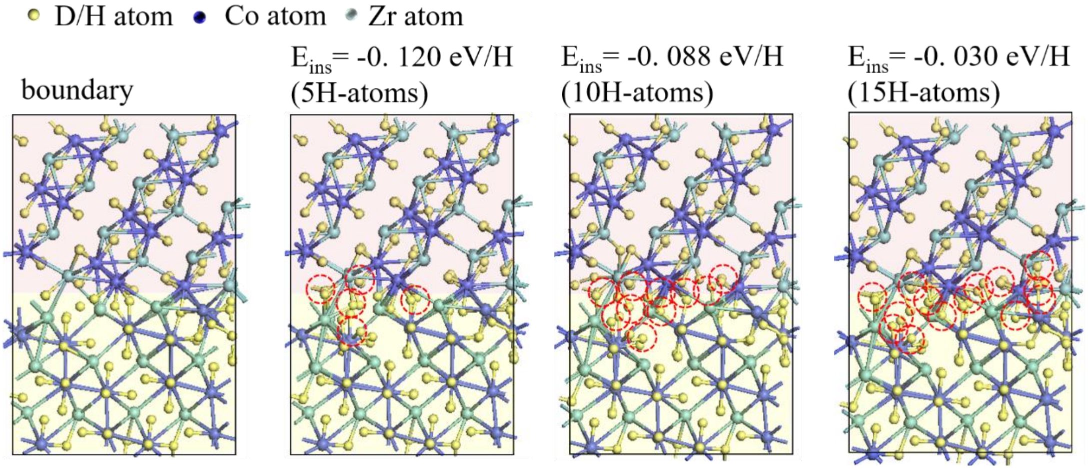

Supplementary Figure 19. Side views of the boundary models of $ZrCoH_3$ with multiple H/D atoms inserted. The red circle indicates the inserted H/D atom. Two different background colors indicate different orientations.

Calculations show that a large number of H atoms can accumulate at the grain boundary. E_f is negative when 15 H atoms are inserted, indicating that the system is thermodynamically stable. This suggests that “trapped-hydrogen” phenomenon exists near the defects, where disproportionate reaction is more likely to occur.

Supplementary Table 4. A summary of E_f of an additional H inserted in the marked positions of the surface/subsurface layers of $ZrCoH_3$.

Position	Energy (eV)
Surface	-1.941
Site 1	0.318
Site 2	-1.179
Site 3	-1.165
Site 4	-0.924
Bulk	0.273

Supplementary Table 5. A summary of E_f of an additional H inserted in the marked positions in the grain boundary of $ZrCoH_3$.

Position	Energy (eV)
Site 1	0.206
Site 2	0.132
Site 3	-0.337
Site 4	-0.389
Site 5	0.092
Site 6	0.111

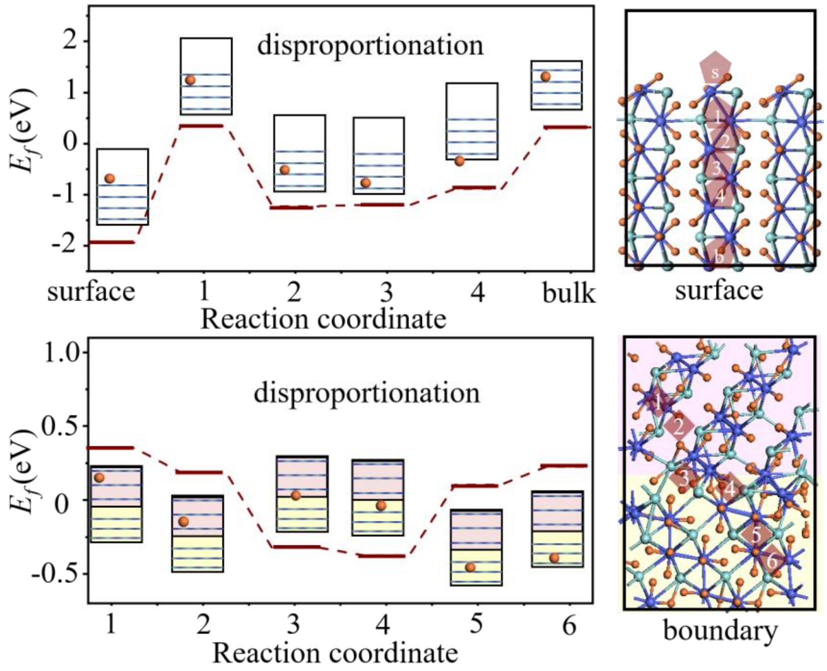

Supplementary Figure 20. E_f of an additional D in the surface/subsurface layers and grain boundary of $ZrCoD_3$. The right sides illustrate the positions of the additional D in the $ZrCoD_3$ model. Two different background colors indicate different orientations. The conclusion is same as that of addition of H in $ZrCoH_3$.

Supplementary Table 6. A summary of E_f of an additional D inserted the marked positions of the surface/subsurface layers of $ZrCoD_3$.

Position	Energy (eV)
Surface	-1.922
Site 1	0.340
Site 2	-1.226
Site 3	-1.201
Site 4	-0.883
bulk	0.319

Supplementary Table 7. A summary of E_f of an additional D inserted the marked positions in the grain boundary of $ZrCoD_3$.

Position	Energy (eV)
Site 1	0.353
Site 2	0.189
Site 3	-0.321
Site 4	-0.375
Site 5	0.104
Site 6	0.226

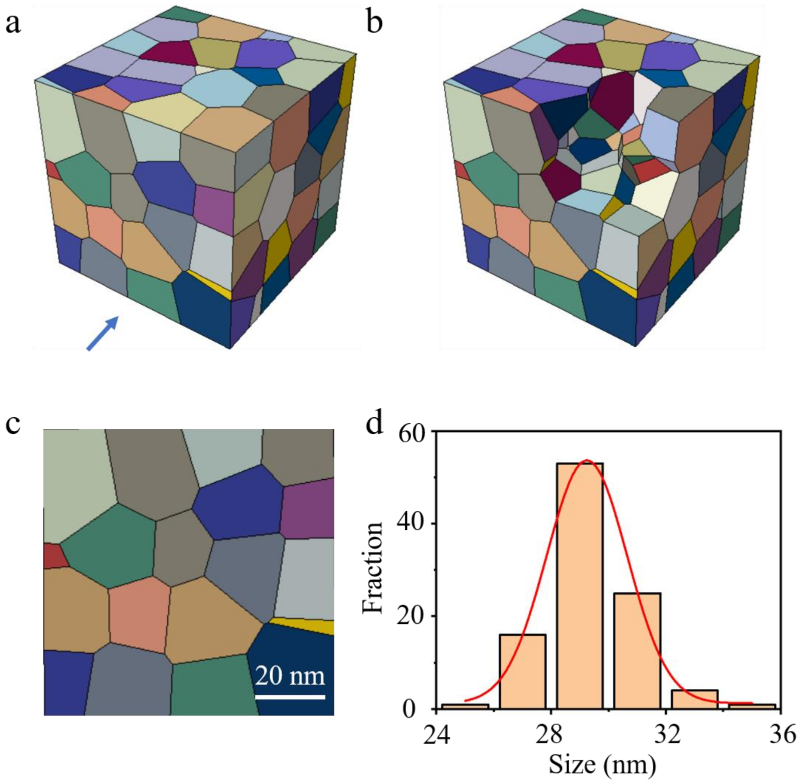

Supplementary Figure 21. (**a, b**) The 3D Voronoi tessellation model. (**c**) Side view of ZC-177μm and (**d**) its crystallite size distribution.

(1) Grain boundary density (ρ_b) of ZC-67nm.

TEM images show that ZC-67nm exhibits quasi-spherical particles. Therefore, we established a spherical model to simulate ZC-67nm. The ρ_b can be calculated as:

$$\rho_b = \frac{S_{\mathrm{sph}}}{V_{\mathrm{sph}}} = \frac{4\pi r^2}{\frac{4}{3}\pi r^3} = \frac{3}{r} \tag{29}$$

where r, S_{sph} and V_{sph} are the radius, surface area and volume of the sphere. Here, r = 34 nm, and the ρ_s value of ZC-67nm is 0.088 nm^{-1}.

(2) Grain boundary density of ZC-177μm.

We established a Voronoi tessellation model to simulate the polycrystalline structure of ZC-177μm, as shown in Supplementary Fig. 21. The Voronoi tessellation diagram was generated by Python, and the model containing the Voronoi tessellation diagram was built using ABAQUS software. The distribution of crystallite size was

controlled by a coefficient that affects the distance between two seeds. We established a model containing 100 grains with an average size of 29 nm (based on the Rietveld refinement results of XRD pattern).

The ρ_b of ZC-177μm can be calculated:

$$\rho_b = \frac{\sum_{100} S_{\mathrm{voro}}}{\sum_{100} V_{\mathrm{voro}}} \tag{30}$$

where S_{voro} and V_{voro} is the surface area and volume of each voro-module, both of which can be obtained statistically. Here, the total S_{voro} and V_{voro} are ~325240.43 nm^2 and ~555,209.56 nm^3, respectively. The ρ_b value of ZC-177μm is 0.586 nm^{-1}.

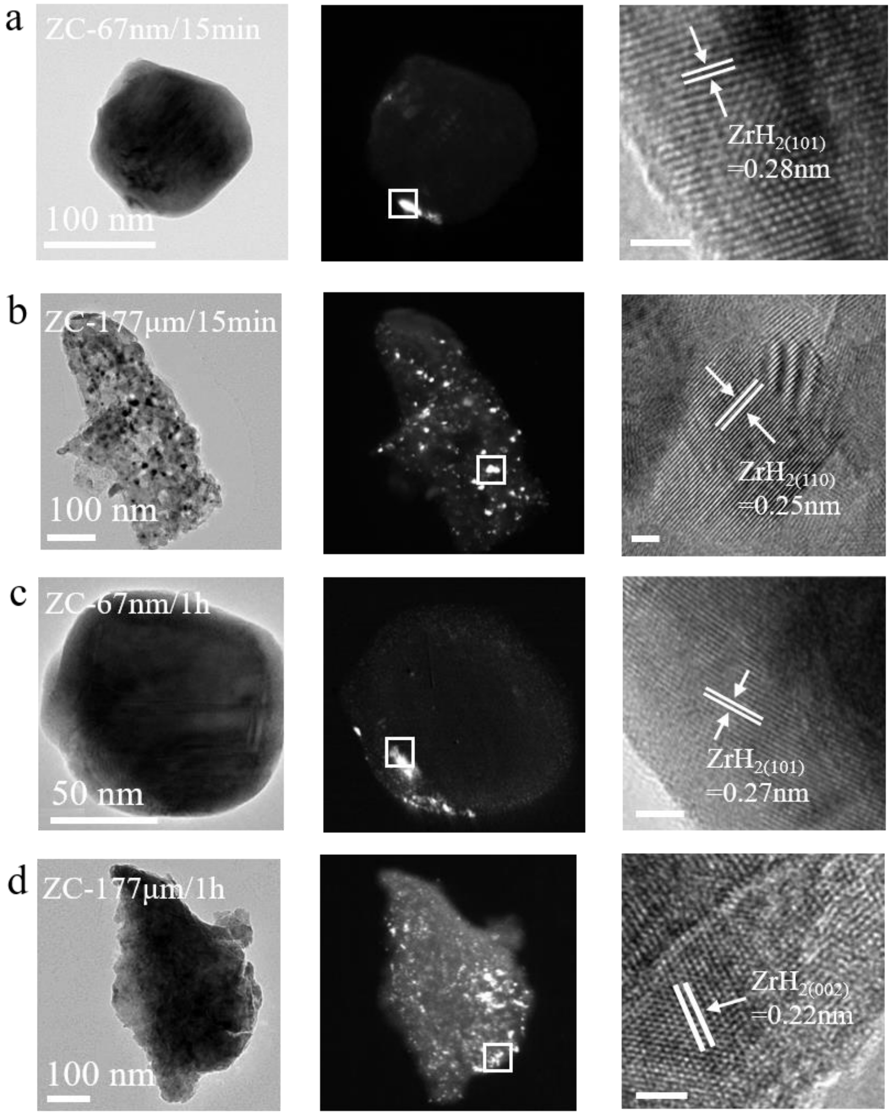

Supplementary Figure 22. Bright/dark-field TEM and HRTEM images of (**a**) ZC-67nm/15min, (**b**) ZC-177μm/15min, (**c**) ZC-67nm/1h, (**d**) ZC-177μm/1h, the scale bars in HRTEM images are 2 nm.

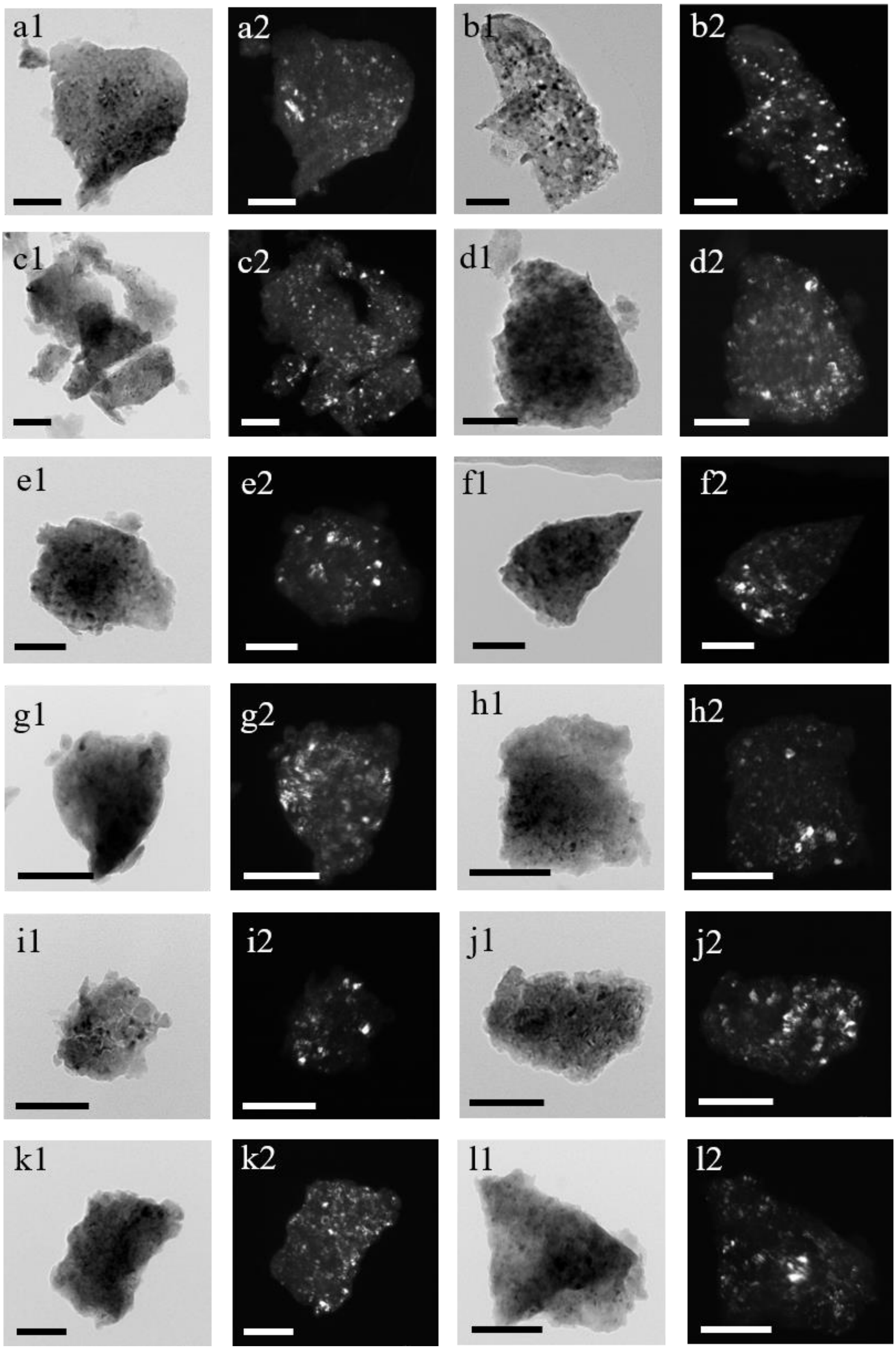

Supplementary Figure 23. Bright/dark-field TEM images of ZC-177μm/15min, the scale bars are 100 nm.

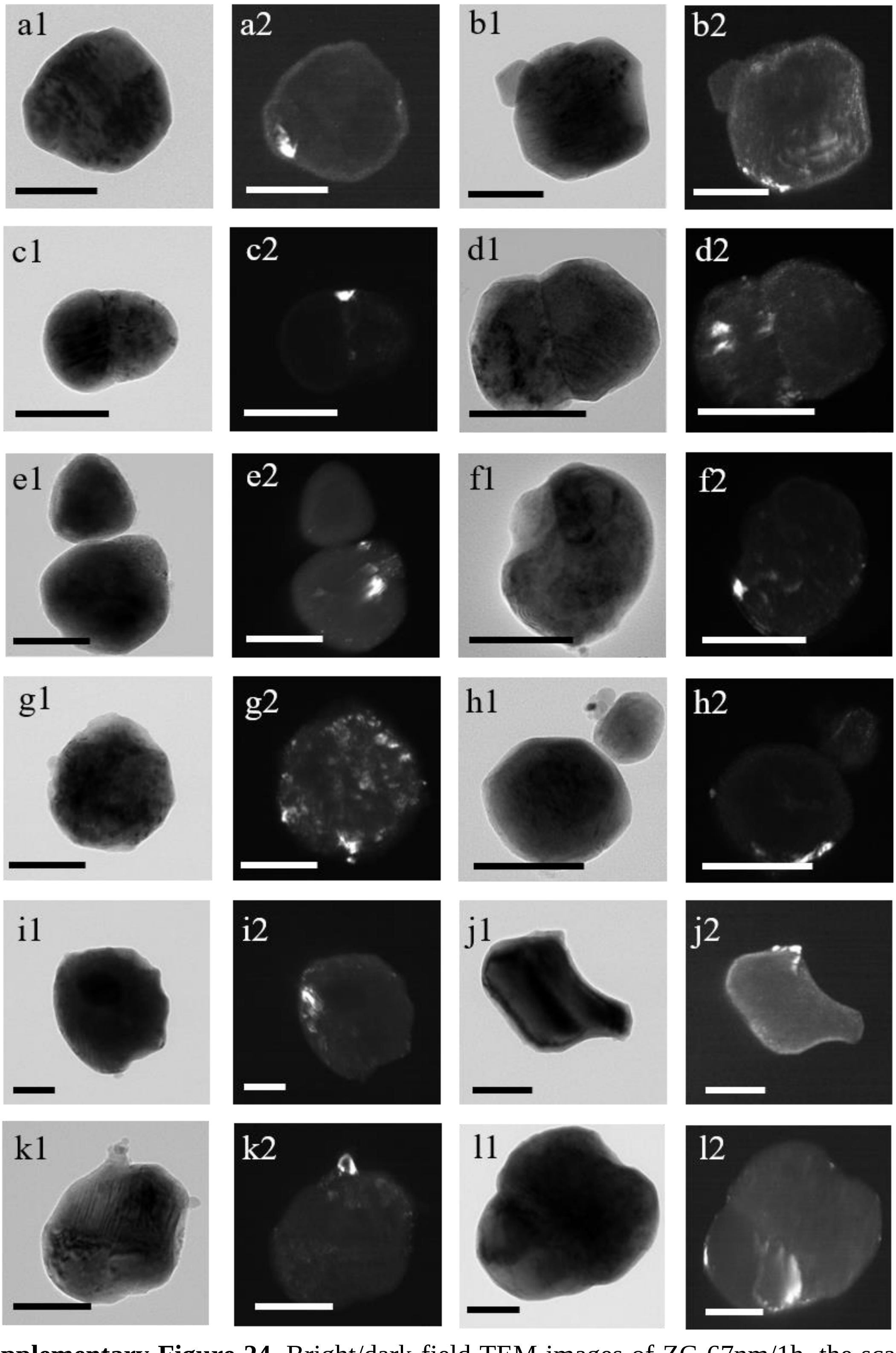

Supplementary Figure 24. Bright/dark-field TEM images of ZC-67nm/1h, the scale bars are 100 nm.

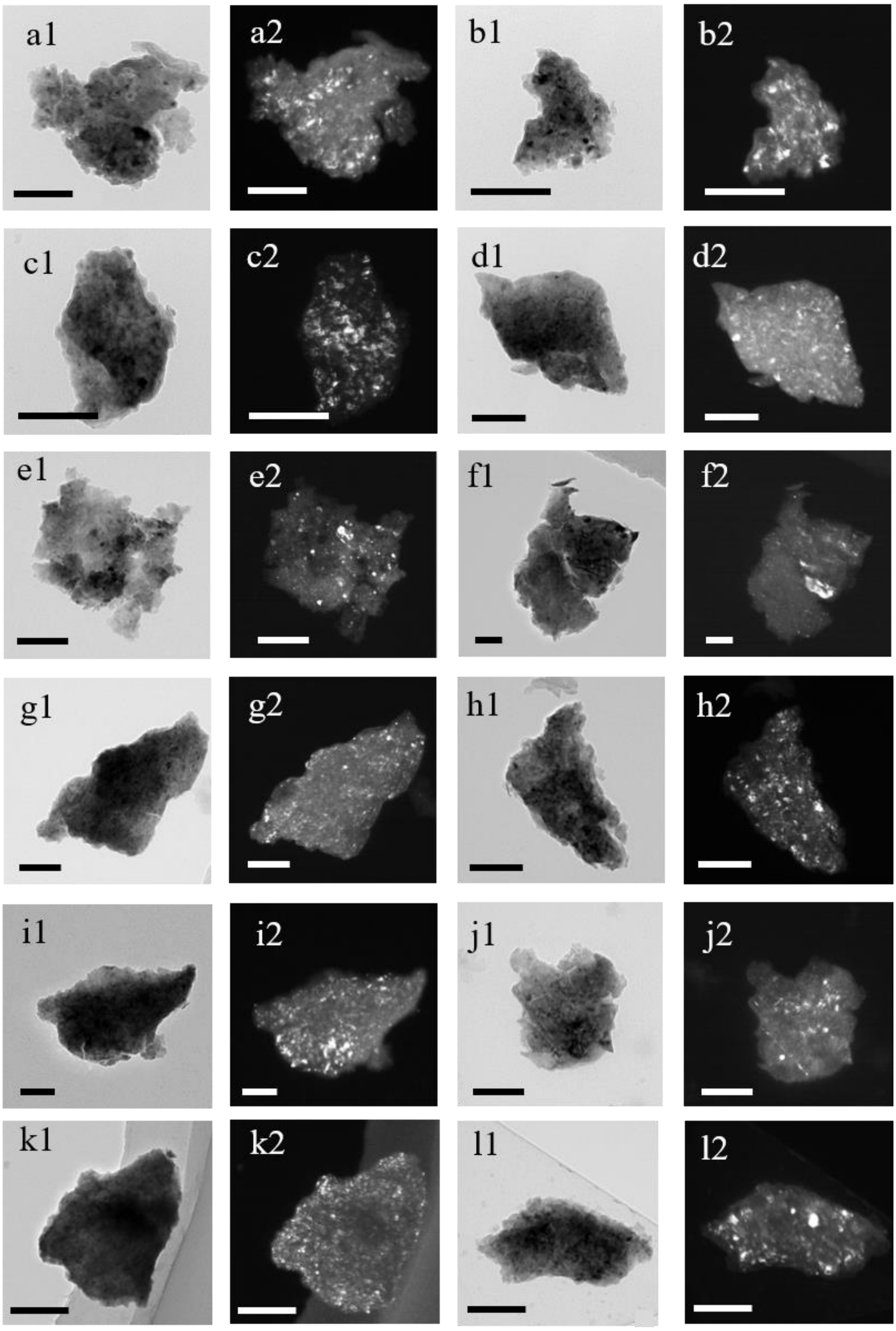

Supplementary Figure 25. Bright/dark-field TEM images of ZC-177μm/1h, the scale bars are 100 nm.

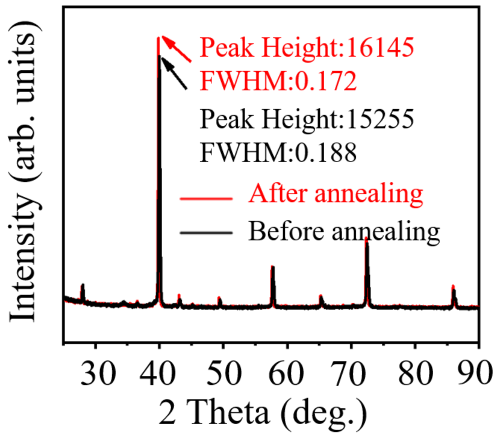

Supplementary Figure 26. XRD patterns of ZC-67nm before and after annealing.

The crystallinity of ZC-67nm was improved after annealing.

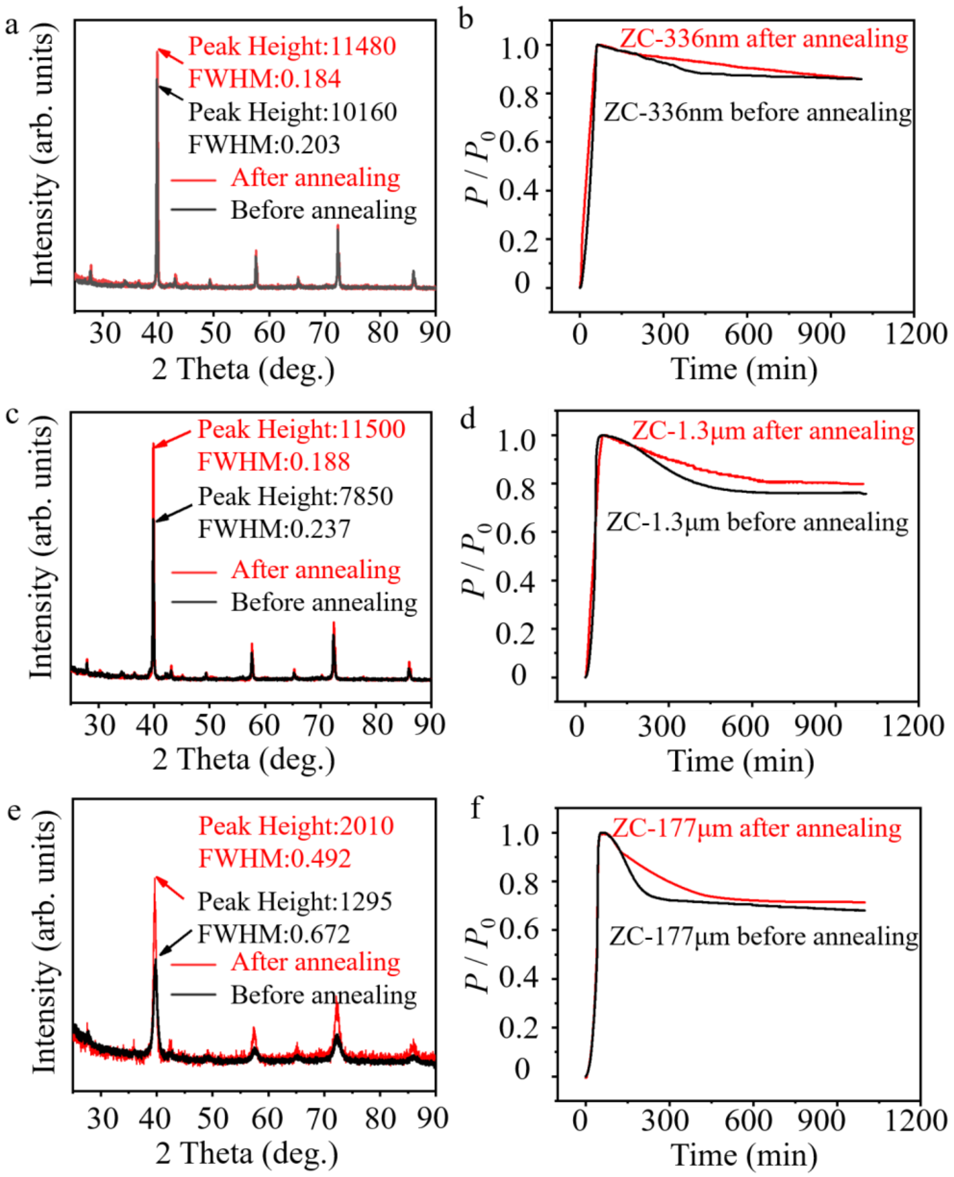

Supplementary Figure 27. XRD patterns of ZrCo before and after annealing, and disproportionation curves of the annealed-ZrCo. (**a, b**) ZC-336nm; (**c, d**) ZC-1.3μm; (**e, f**) ZC-177μm.

After annealing, ZrCo alloys show the improved crystallinity and the decreased disproportionation ratio, indicating that crystal defects are the cause of the disproportionation reaction.

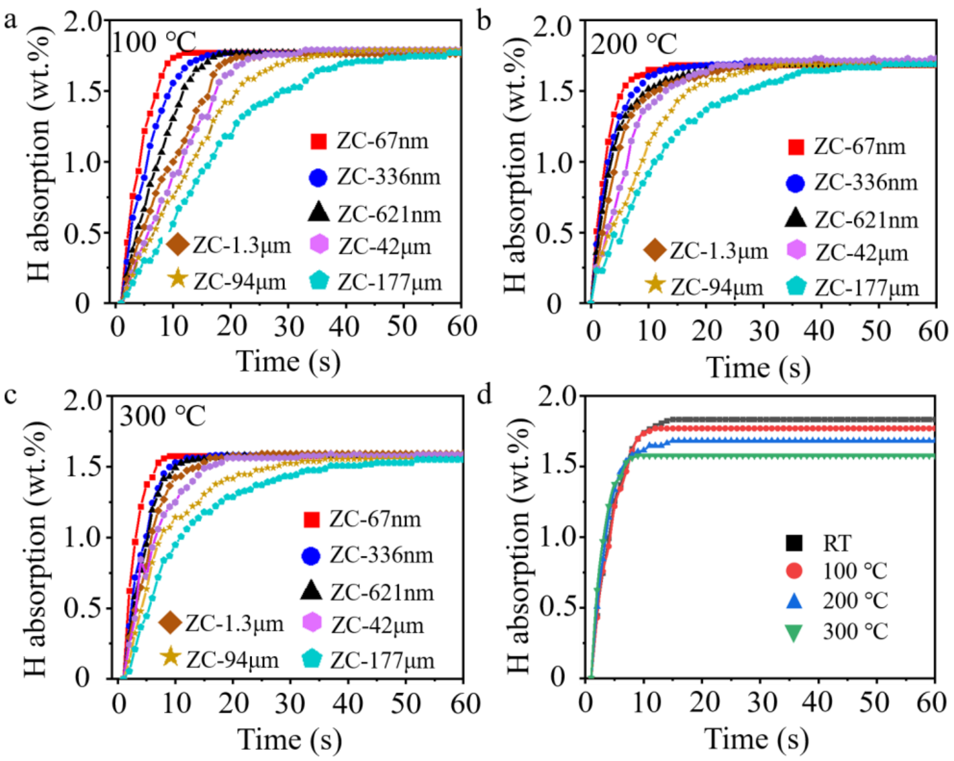

Supplementary Figure 28. The isothermal hydrogenation curves of the activated ZrCo alloys at (**a**) 100 ℃, (**b**) 200 ℃ and (**c**) 300 ℃. (**d**) Comparison of isothermal hydrogenation curves of ZC-67nm at different temperatures.

The hydrogen absorption rate of the activated ZrCo alloys increases with the decrease of particle size.

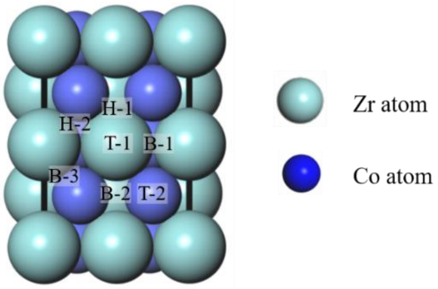

Supplementary Figure 29. Top view of ZrCo (110) surface. Possible H inserted sites on the (110) surface of ZrCo, including Top (T-1, T-2), Bridge (B-1, B-2, B-3), Hollow (H-1, H-2) types.

It has been reported that (110) is the most stable facet of ZrCo[18-20], which is also proved by our XRD results (Supplementary Fig. 12). So (110) was chosen to investigate the adsorption and diffusion of H atoms in ZrCo. Combined with the previous calculation[18], the octahedral interstitial H-1 is the most stable position in ZrCo (110), so H-1 was chosen as the hydrogen/deuterium position.

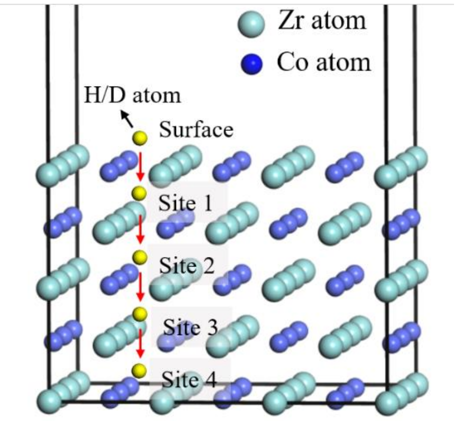

Supplementary Figure 30. Diffusion path of H/D adsorbed on ZrCo (110) surface.

Chattaraj et al.[15] proved that H atoms tend to diffuse vertically along the H-1 adsorption location because of the lowest energy barrier. Therefore, we chose a vertical diffusion path for H/D from the surface to site 4.

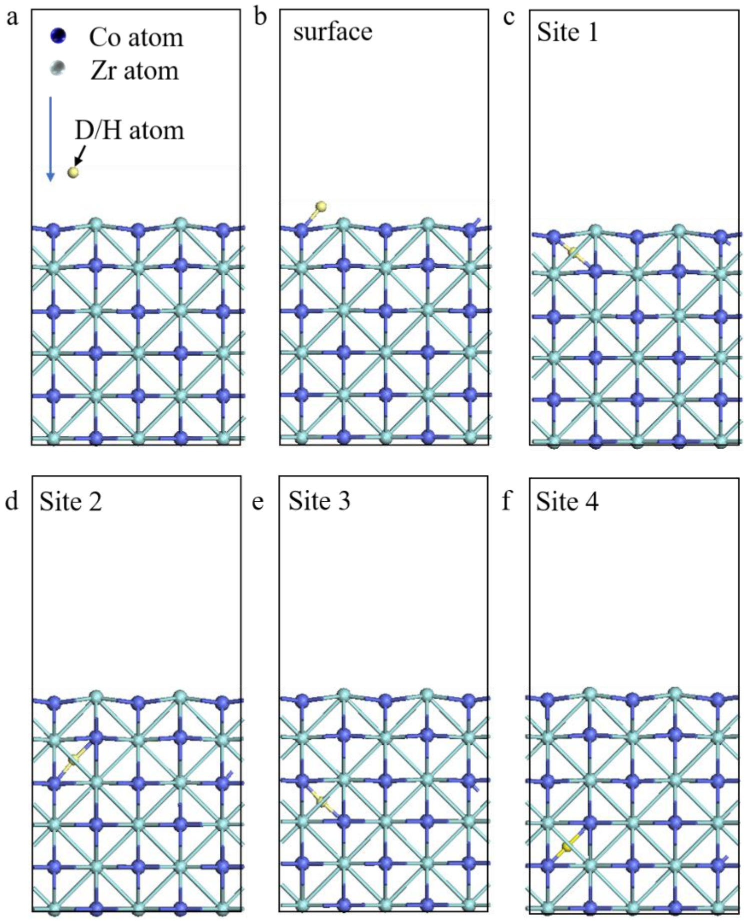

Supplementary Figure 31. Side views of H/D adsorption through ZrCo(110). (**a**) H/D atom and ZrCo(110) surface/subsurface layers, (**b**) H/D adsorption on ZrCo(110) surface, (**c-f**) H/D insertion into octahedral interstitials of the subsurface layers.

Supplementary Table 8. A summary of E_f of H_i/D_i at different positions. The data is extracted from Fig. 3b.

Position	Energy (eV)	
	Hydrogen	Deuterium
Surface	-0.776	-0.834
Site 1	0.457	0.463
Site 2	-0.233	-0.190
Site 3	-0.206	-0.159
Site 4	-0.214	-0.228

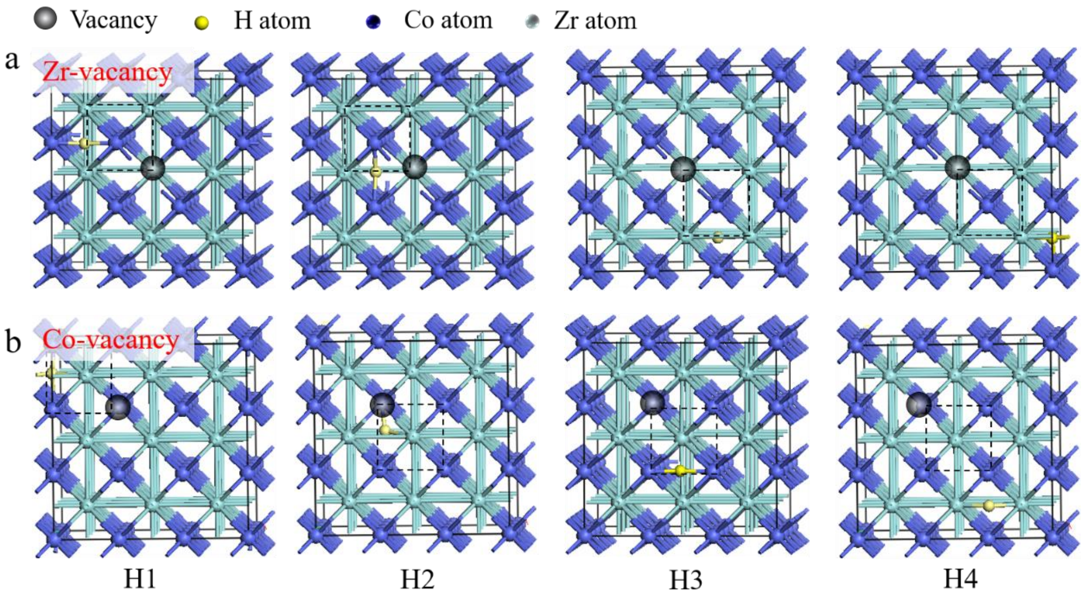

Supplementary Figure 32. H diffusion paths around (**a**) Zr vacancy and (**b**) Co vacancy in ZrCo.

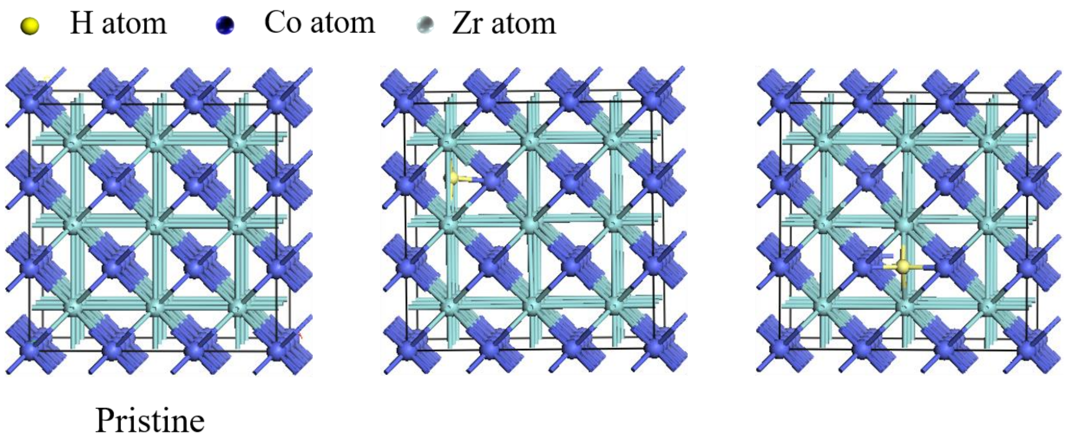

Supplementary Figure 33. E_f of H_i in non-defective ZrCo (Fig. 3c, H5 site). The E_f is 0.303 eV.

Supplementary Table 9. A summary of E_f of H_i in ZrCo without (H5 site) and with defects (H1-H4 sites). The data is extracted from Fig. 3c.

**Position**	**Energy (eV)**	
	**Zr vacancy**	**Co vacancy**
H1	-0.279	-0.208
H2	-0.403	-0.395
H3	-0.253	-0.185
H4	-0.293	-0.277
H5	-0.303	-0.303

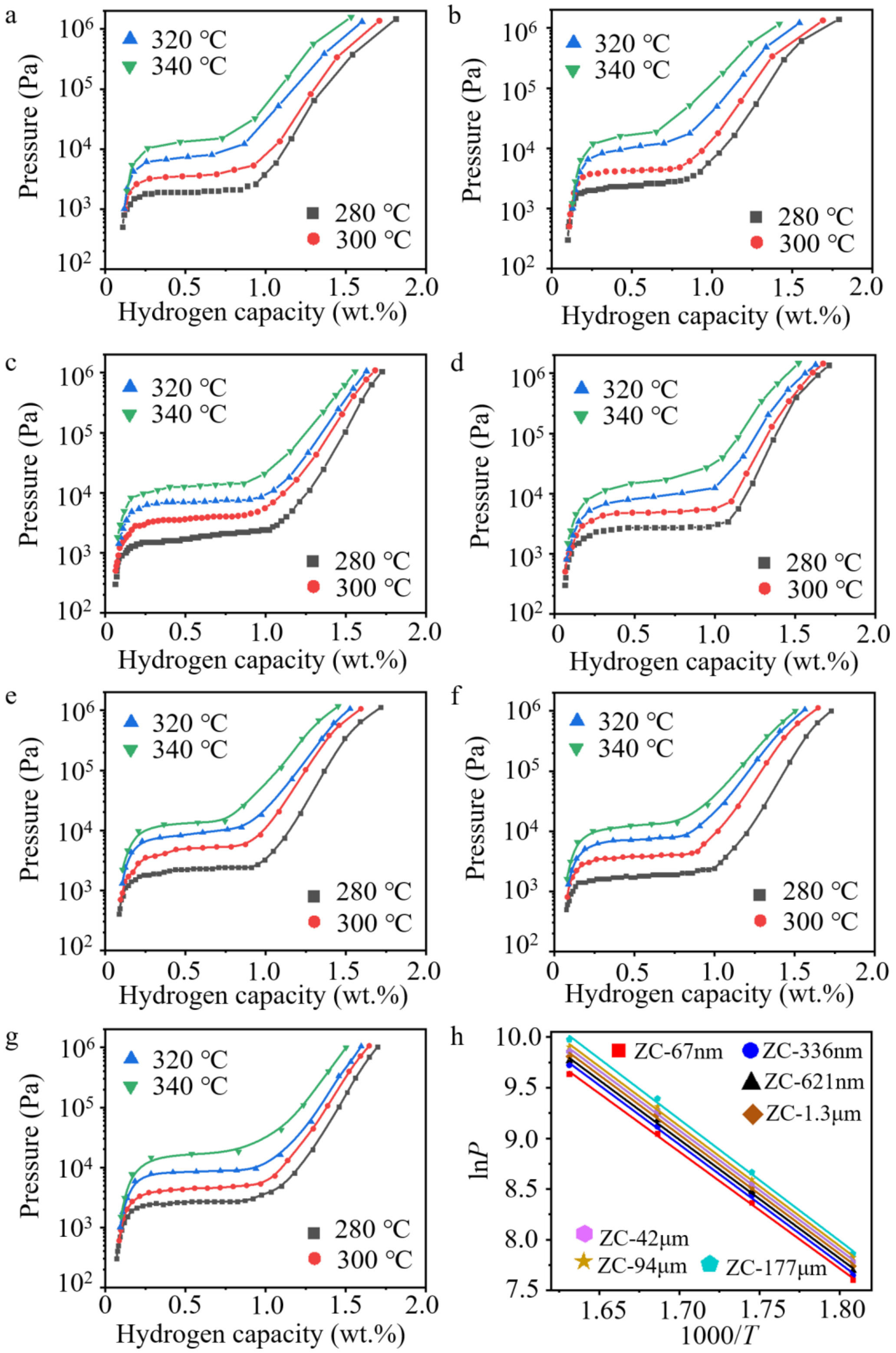

Supplementary Figure 34. PCT curves of (**a**) ZC-67nm; (**b**) ZC-336nm; (**c**) ZC-621nm; (**d**) ZC-1.3μm; (**e**) ZC-42μm; (**f**) ZC-94μm; (**g**) ZC-177μm. (**h**) Van't Hoff plots of the above samples.

Supplementary Table 10. A summary of enthalpy (Δ*H*) and entropy (Δ*S*) values of ZrCo alloys. The data is extracted from Supplementary Fig. 34.

**Sample**	**Δ*H* (kJ mol^{-1})**	**Δ*S* (J mol^{-1} K^{-1})**
ZC-67nm	95.7±1.9	236.5±3.2
ZC-336nm	97.3±1.8	239.9±3.2
ZC-621nm	97.5±2.3	240.4±3.9
ZC-1.3μm	97.8±2.7	241.3±4.6
ZC-42μm	98.0±2.4	242.0±4.1
ZC-94μm	98.2±2.1	242.7±3.6
ZC-177μm	99.7±2.8	245.9±4.8

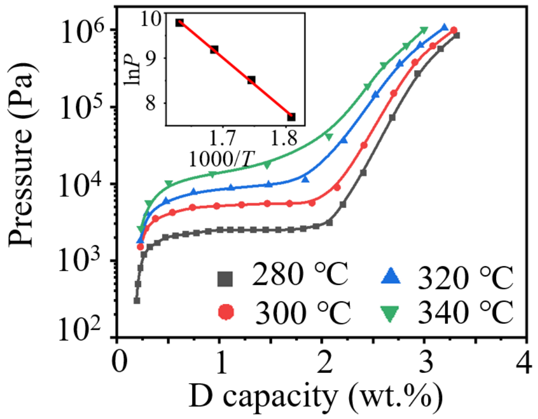

Supplementary Figure 35. PCT curves of ZC-67nm-D. Inset: Van't Hoff plot.

The ΔH and ΔS values for deuterium desorption were calculated to be 98.0 kJ mol^{-1} and 241.5 J mol^{-1} K^{-1}, respectively. These values are higher than those of hydrogen desorption (95.7 kJ mol^{-1} and 236.5 J mol^{-1} K^{-1}), indicating that ZC-67nm has an obvious isotope effect.

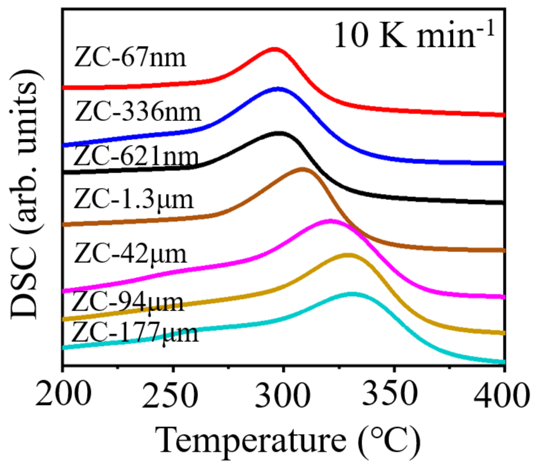

Supplementary Figure 36. DSC curves of ZrCo alloys. The heating rate is 10 K min^{-1}.

The DSC curves were collected to characterize the hydrogen release process. With the decrease of particle size, the peak temperature decreases from 331.2 to 295.8 °C. The peak temperature is reduced by almost 40 °C.

Supplementary Table 11. A summary of TPD peaks (T_{TPD}), DSC peaks (T_{DSC}). The data is extracted from Fig. 3e and Supplementary Fig. 36.

Sample	T_{TPD}/°C	T_{DSC}/°C
ZC-67nm	281.1	295.8
ZC-336nm	284.3	297.3
ZC-621nm	286.9	297.8
ZC-1.3μm	294.4	308.8
ZC-42μm	309.1	321.5
ZC-94μm	315.2	328.9
ZC-177μm	336.8	331.2

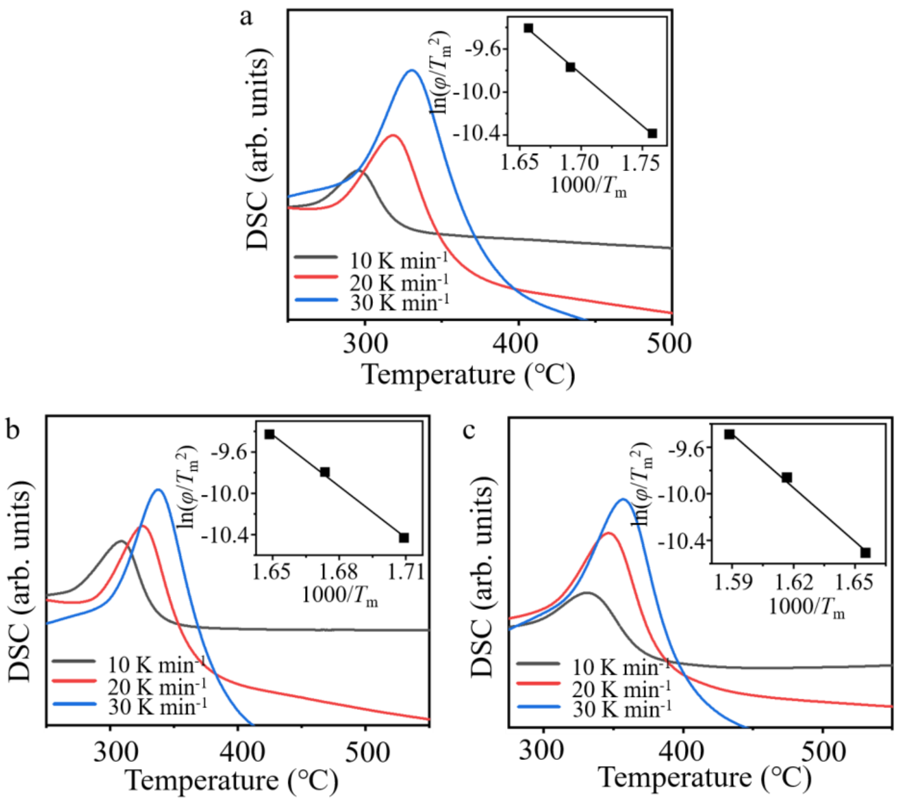

Supplementary Figure 37. DSC curves and Kissinger plots of (**a**) ZC-67nm, (**b**) ZC-1.3μm, (**c**) ZC-177μm with heating rates of 10, 20 and 30 K min^{-1}, respectively.

Dehydrogenation activation energies (E_{de}) of ZC-67nm, ZC-1.3μm and ZC-177μm were calculated to be 80.1, 103.8, and 127.9 kJ mol^{-1}, respectively. The E_{de} of the chem-ZrCo is significantly lower than that of the smelting-ZrCo.

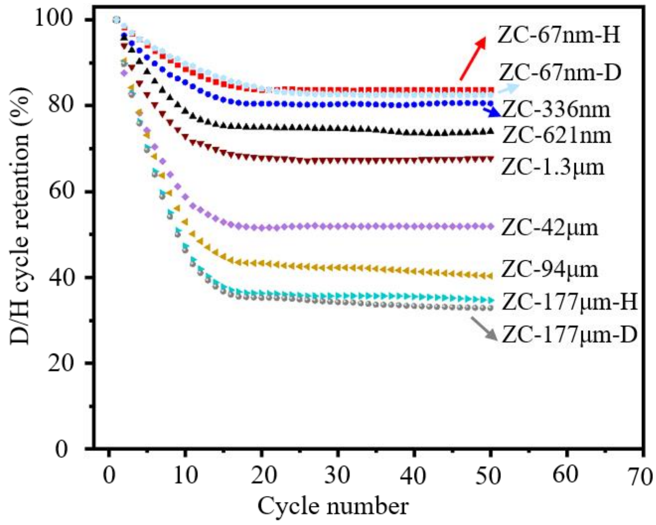

Supplementary Figure 38. The cyclability of as-synthesized ZrCo alloys.

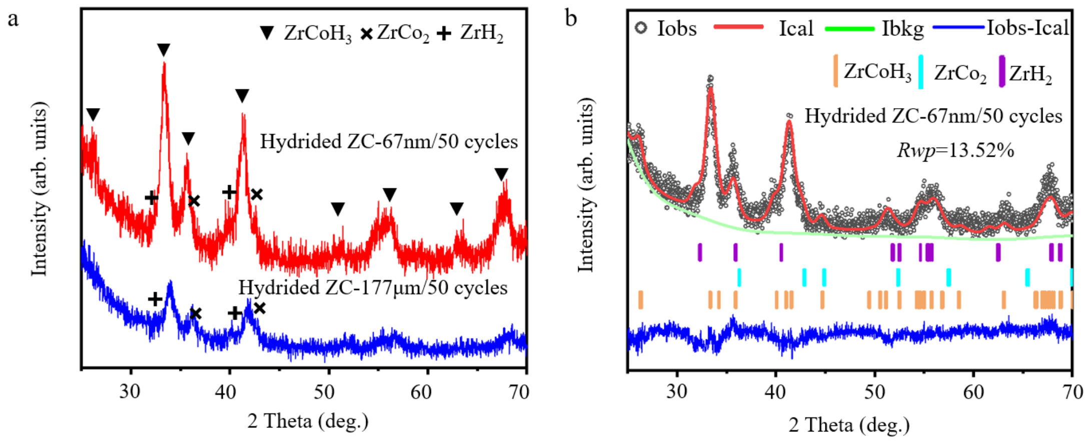

Supplementary Figure 39. (**a**) XRD patterns of hydrided ZC-67nm/50 cycles and ZC-177μm/50 cycles. (**b**) XRD patterns and Rietveld refinement results of hydrided ZC-67nm/50 cycles.

Supplementary Table 12. Rietveld refinement results of XRD patterns of hydrided ZC-67nm/50 cycles.

**Sample**	**Lattice parameters (Å)**			**Volume (Å³)**	**Phase abundance (wt.%)**		
	**a**	**b**	**c**		**$ZrCoH_3$**	**$ZrCo_2$**	**ZrH_2**
ZC-67nm/50 cycles	3.5317	10.4758	4.3487	160.893	90.4	5.6	4.0

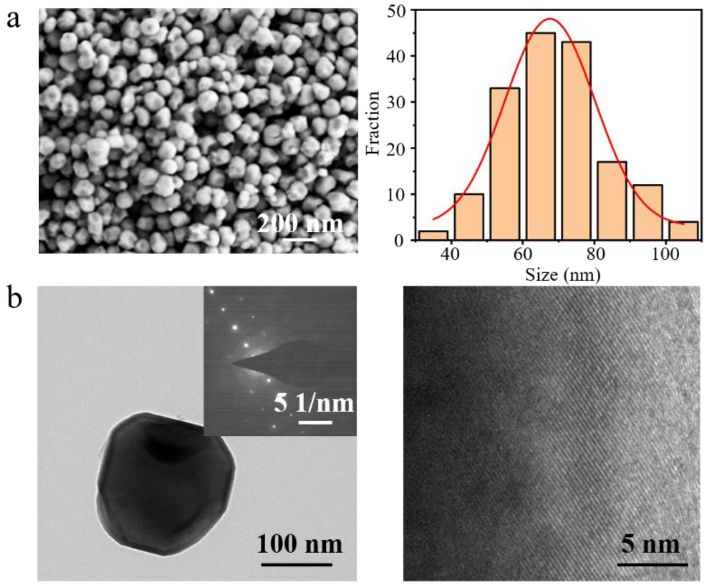

Supplementary Figure 40. (**a**) SEM image of ZC-67nm after 50 cycles and the distribution of particle size. (**b**) TEM, SAED and HRTEM of ZC-67nm after 50 cycles.

ZC-67nm after 50 cycles are still monodisperse with an average size of ~67 nm. No obvious agglomeration and crystal growth were observed.

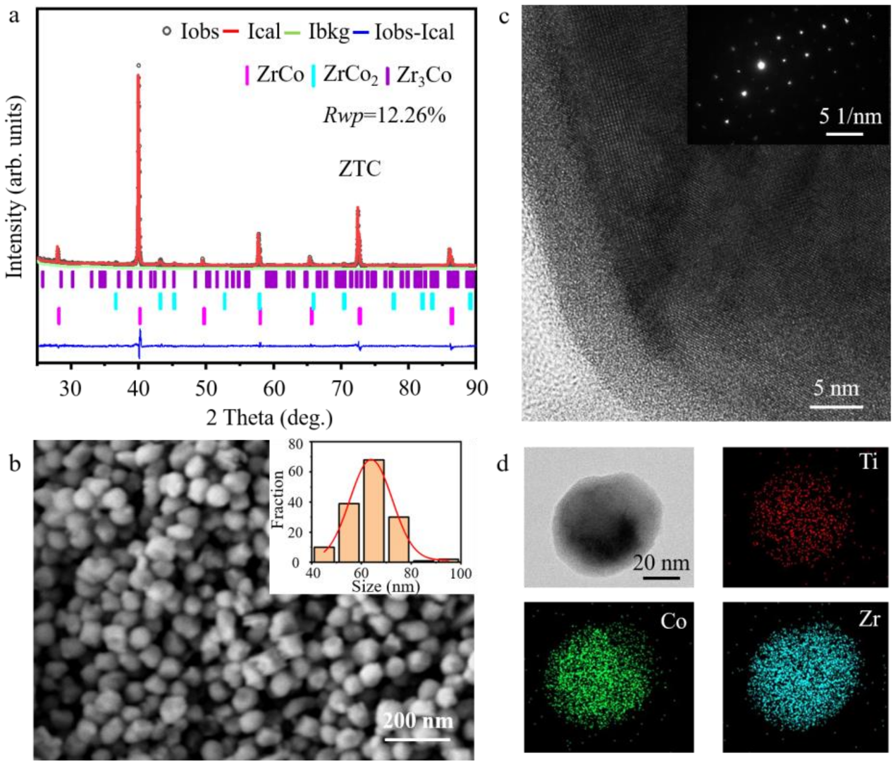

Supplementary Figure 41. Structure and morphology of ZTC alloy. (**a**) XRD pattern and Rietveld refinement result. (**b**) SEM, (**c**) SAED and HRTEM image, and (**d**) elemental mappings.

The XRD diffraction peaks of ZTC shift towards larger angles compared with ZC-67nm (from Fig. 1b), which is attributed to the shrinkage of the cell volume after Ti substitution.

Supplementary Table 13. Rietveld refinement result of XRD pattern of ZTC.

Sample	Lattice constant of ZrCo phase (Å)	Cell volume of ZrCo phase (Å^3)	Phase abundance (wt.%)		
			ZrCo	$ZrCo_2$	Zr_3Co
ZTC	3.178	32.081	95.2	3.1	1.7

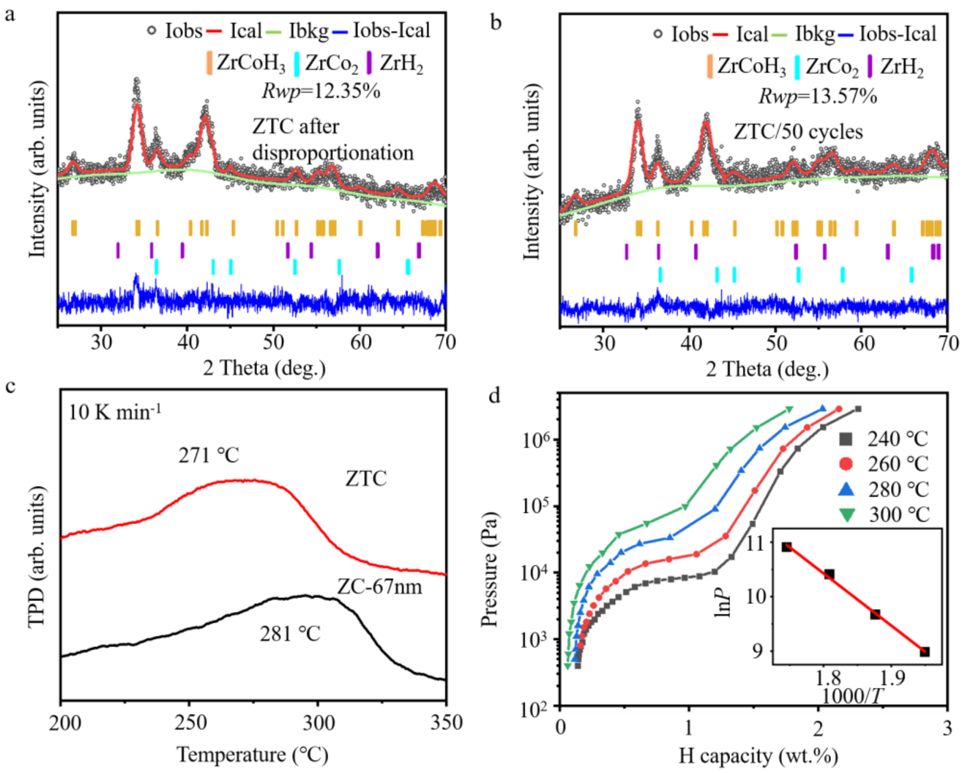

Supplementary Figure 42. Rietveld refinement results of XRD patterns of (**a**) ZTC after disproportionation and (**b**) hydrided ZTC/50 cycles. (**c**) TPD curves. (**d**) PCT curves of ZTC. Inset: Van't Hoff plot.

Supplementary Table 14. Rietveld refinement results of XRD patterns of ZTC after disproportionation and 50 cycles.

**Sample**	**Phase abundance (wt.%)**		
	**$ZrCoH_3$**	**$ZrCo_2$**	**ZrH_2**
ZTC after disproportionation	93.9	3.5	2.6
hydrided ZTC/50 cycles	92.3	5.5	2.2

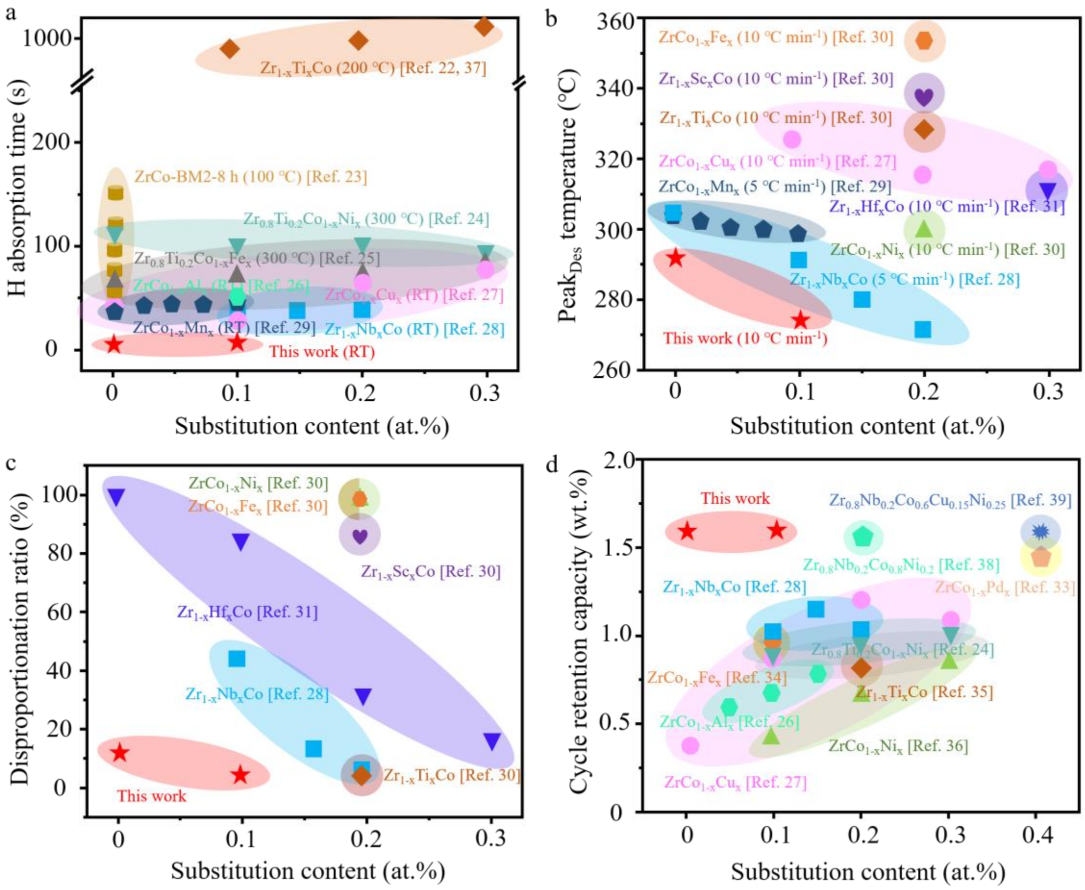

Supplementary Figure 43. Comparisons of hydrogen storage performances of ZC-67nm and ZTC with other ZrCo alloys reported in literatures[22-39]. (**a**) Hydrogenation time to reach 90% of the maximum capacity. (**b**) DSC peak temperatures of dehydrogenation process. (**c**) Disproportionation ratio collected at 500 °C. (**d**) Cyclability.

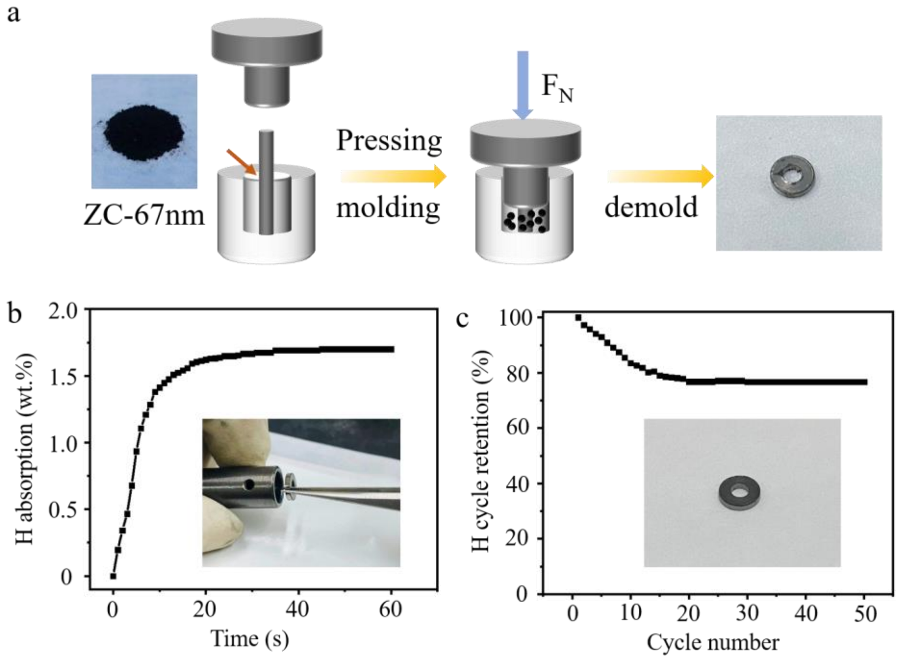

Supplementary Figure 44. (**a**) Schematic illustration of the preparation of millimeter-scale ZrCo ring. (**b**) Hydrogenation kinetics of the ring. Inset: the photo image of the ring and sample tube of Sieverts' PCT apparatus. (**c**) The cyclability of ZC-67nm ring. Inset: the photo image of the ring after 50 cycles.

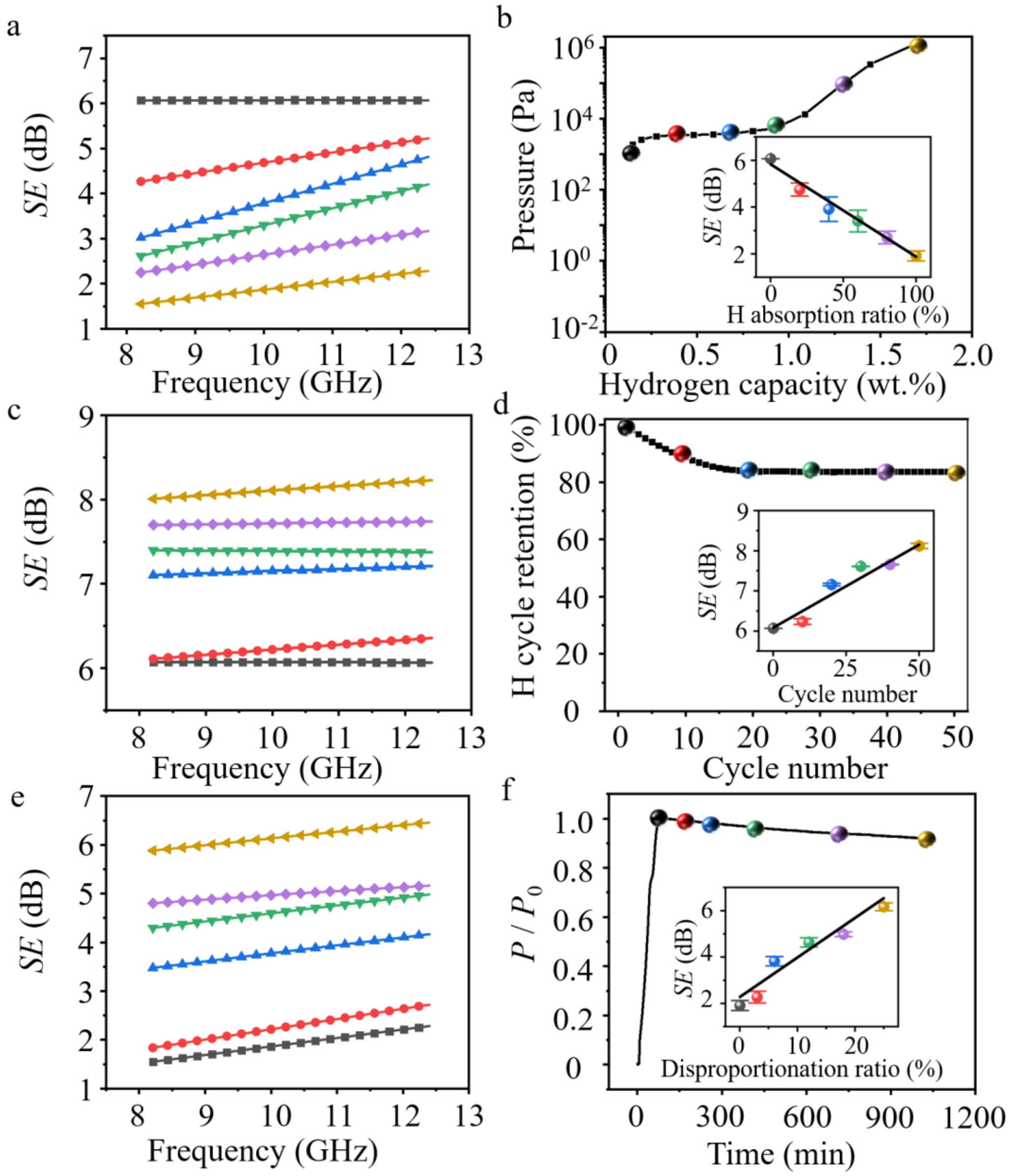

Supplementary Figure 45. Variations of *SE* of ZC-67nm with (**a, b**) hydrogen storage capacity; (**c, d**) cycle number; (**e, f**) disproportionation ratio.

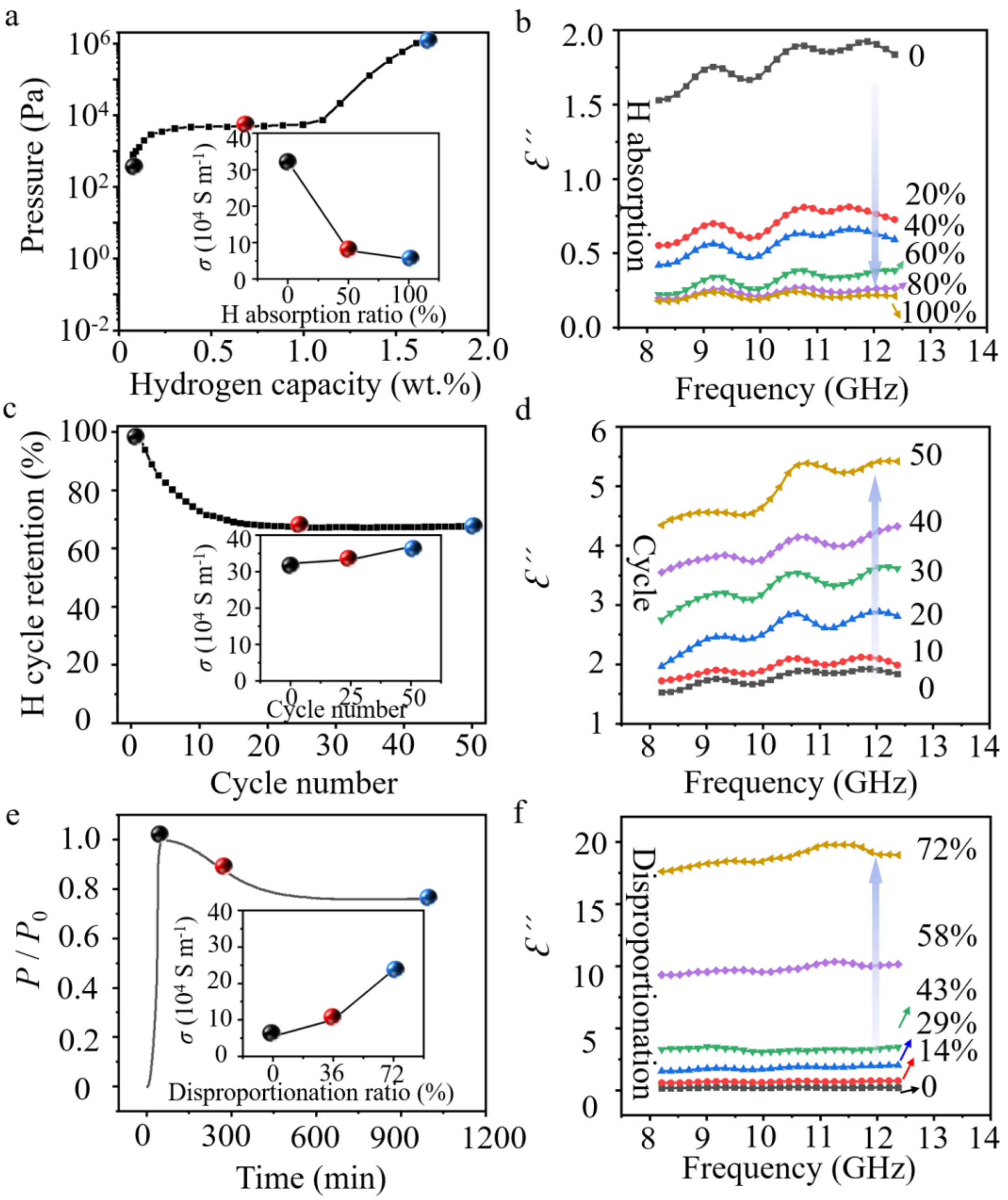

Supplementary Figure 46. Changes of electrical conductivity and imaginary part of permittivity of chem-ZrCo: (**a, b**) hydrogen absorption; (**c, d**) cycle number; (**e, f**) disproportionation process.

Generally, *SE* value increases with conductivity and polarization relaxation of the material[40,41]. An increase in conductivity can increase the reflection of microwaves at the air-sample interface (due to impedance mismatch) and enhance the attenuation of microwaves (i.e. microwave absorption) inside the sample through conduction loss (conversion of electromagnetic energy into thermal energy by induced current)[42]. The increase in polarization relaxation can also enhance microwave attenuation inside the sample (conversion of electromagnetic energy into thermal energy through the rotation

of dipoles)[43]. Heterogeneous interface is an important source of polarization relaxation. Under an alternating electric field (electric field component of microwave), the free charge carriers will accumulate at the heterogeneous interface of two phases and produce space-charge polarization relaxation[44], thus attenuating the electromagnetic energy.

The complex permittivity of a material can be expressed as $\varepsilon=\varepsilon'-i\varepsilon''$. The imaginary part of complex permittivity (ε'') represents the attenuation capability of electromagnetic energy inside the material[45]. According to the Debye theory[46], ε'' consists of conduction loss (ε_c'') and polarization relaxation loss (ε_p''), which follows:

$$\varepsilon''=\varepsilon_c''+\varepsilon_p''=\frac{\sigma}{\omega\varepsilon_0}+\frac{\varepsilon_s-\varepsilon_\infty}{1+\omega^2\tau^2}\omega\tau \tag{31}$$

where ω is the microwave frequency; τ is the polarization relaxation time; ε_s and ε_∞ are the static permittivity and the relative permittivity at the high-frequency limit, respectively; σ is the electrical conductivity.

As shown in Supplementary Fig. 46a-b, the conductivity of ZrCo decreases from 32.3×10^4 to 5.5×10^4 S m^{-1} as the hydrogen storage capacity increases from 0 to 100%. On the one hand, the decrease of conductivity weakens the reflection of microwaves at the air-sample interface. On the other hand, the decrease of conductivity reduces the conduction loss (ε_c''), thus decreasing the attenuation of microwaves inside the material. Due to the decrease of reflection and absorption of microwaves, more microwaves can pass through the sample, i.e. decreasing the electromagnetic interference shielding effectiveness (SE).

As shown in Supplementary Fig. 46c-d, the conductivity of ZrCo increases after 50 cycles, which implies an increase of conduction loss (ε_c''). Moreover, with the increase of cycle number, the content of impurity phases in ZrCo continuously increases, leading to the formation of more heterogeneous interfaces. As a result, the polarization relaxation loss (ε_p'') increases. The increase of ε'' (i.e. $\varepsilon_c''+\varepsilon_p''$) suggests the increase of microwave attenuation, which leads to the enhancement of shielding effectiveness.

As shown in Supplementary Fig. 46e-f, the conductivity of ZrCo significantly increases as the disproportionation ratio increases. Firstly, the increase of conductivity enhances the reflection of microwaves at the air-sample interface. Secondly, the increase of conductivity increases the conduction loss (ε_c''). Thirdly, due to the formation of disproportionation phases, a large number of heterogeneous interfaces form, which increases the polarization relaxation loss (ε_p''). As a result, the reflection and absorption of microwaves increases, i.e. enhancing the shielding effectiveness.

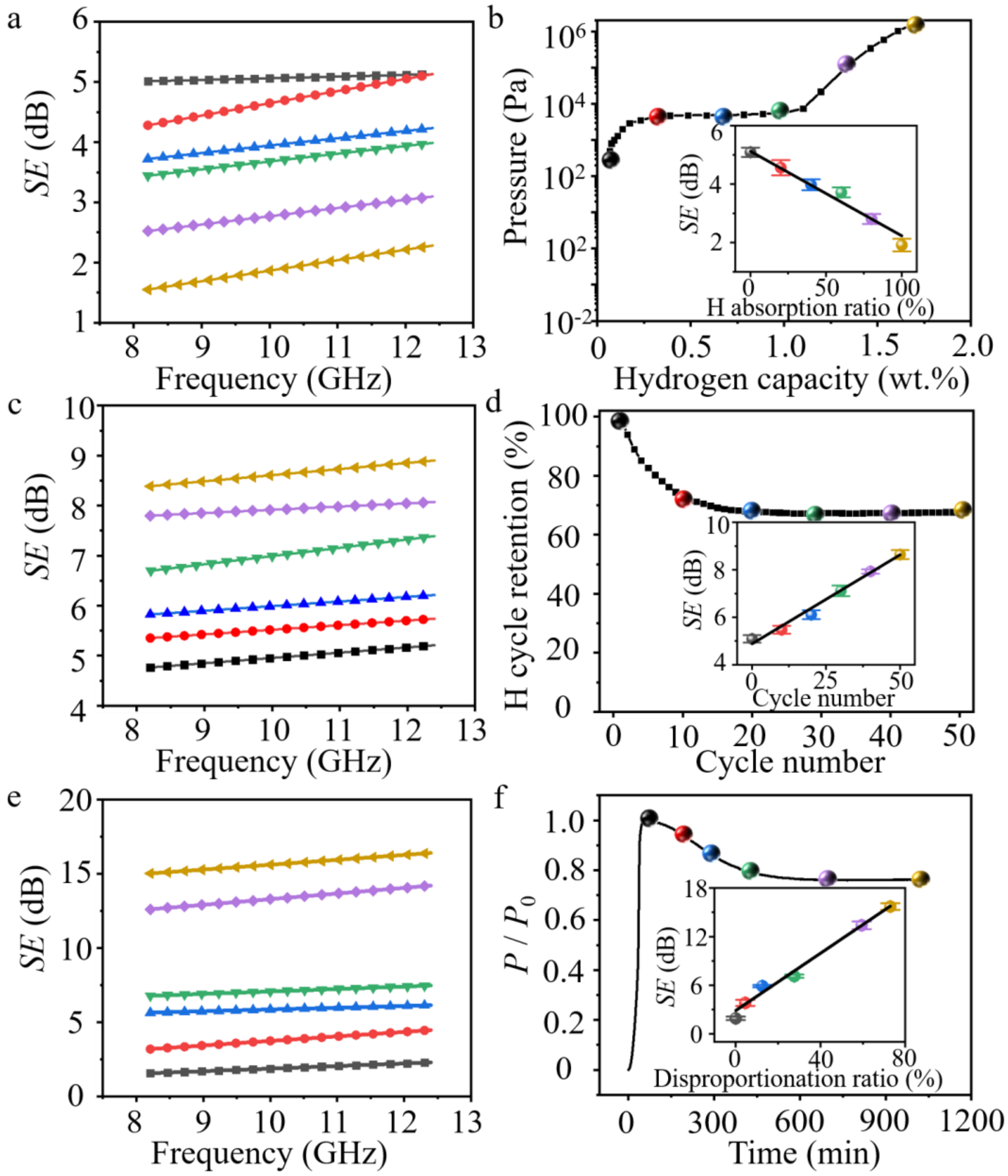

Supplementary Figure 47. Variations of *SE* of ZC-1.3μm with (**a, b**) hydrogen storage capacity; (**c, d**) cycle number; (**e, f**) disproportionation ratio.

The above *SE* curves of ZC-1.3μm were repeatedly tested three times (Supplementary Figs. 47-49) to ensure the repeatability of the data.

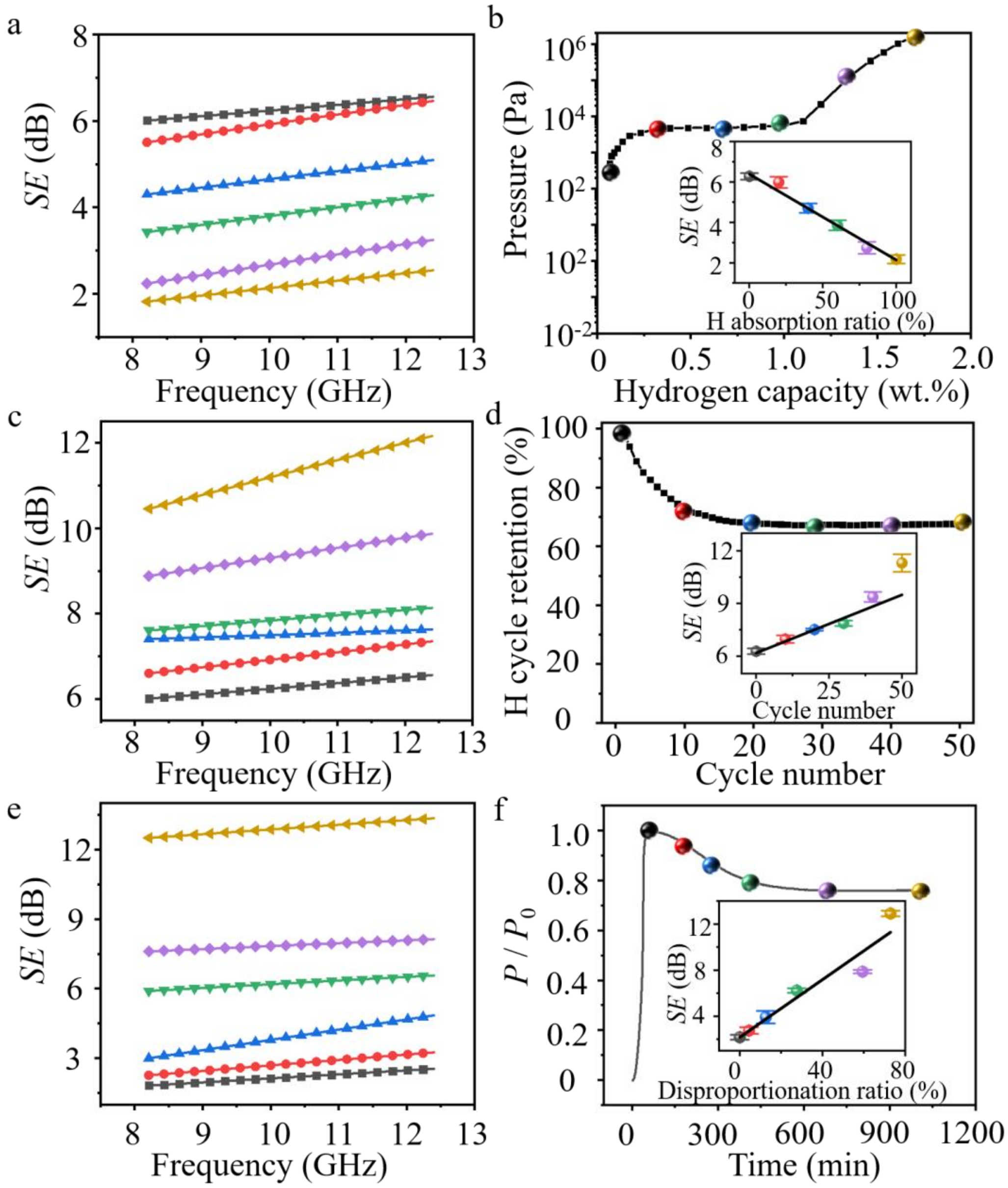

Supplementary Figure 48. Repeated test of ZC-1.3μm. Variations of *SE* with (**a, b**) hydrogen storage capacity; (**c, d**) cycle number; (**e, f**) disproportionation ratio.

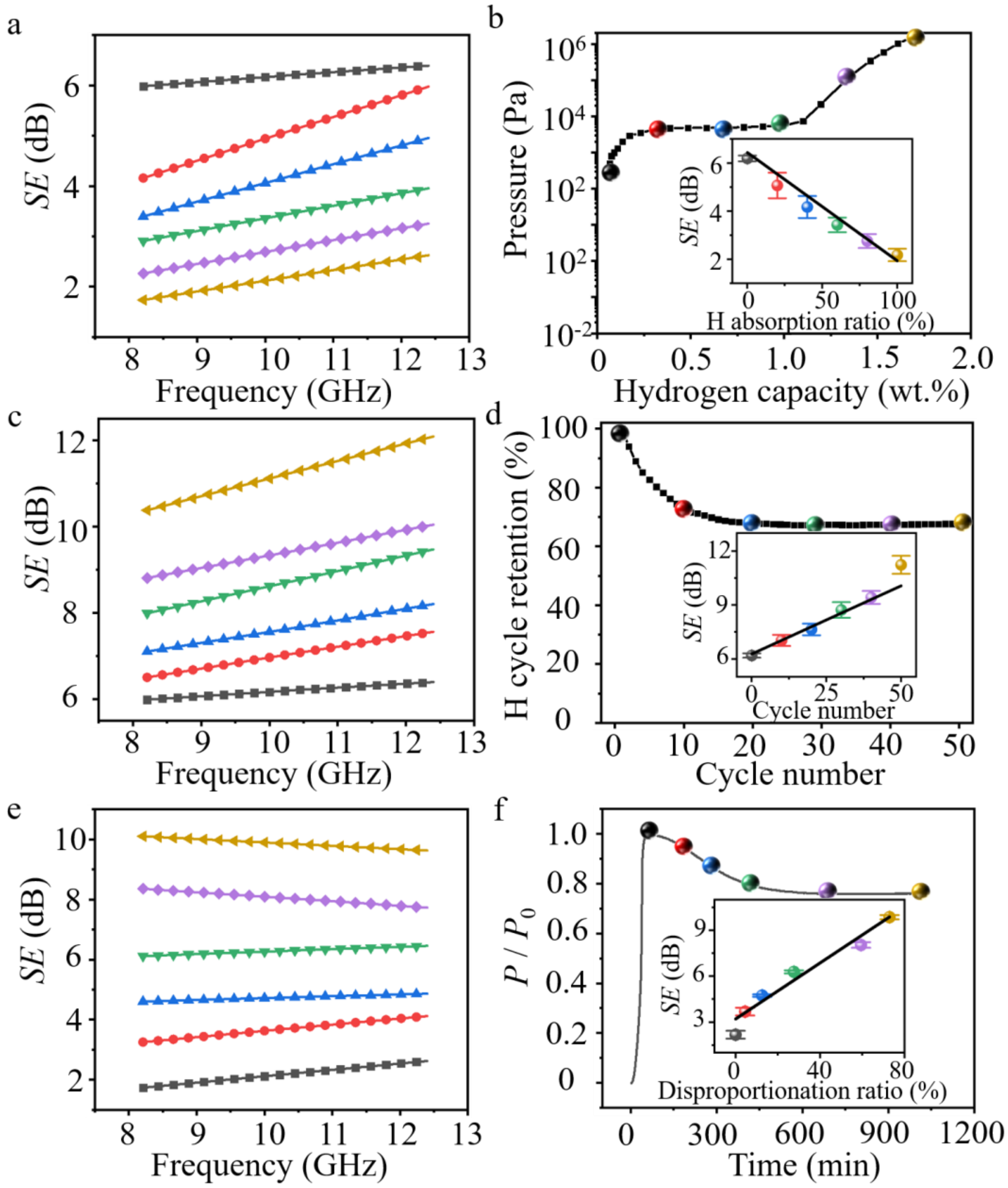

Supplementary Figure 49. Repeated test of ZC-1.3μm. Variations of *SE* with (**a, b**) hydrogen storage capacity; (**c, d**) cycle number; (**e, f**) disproportionation ratio.

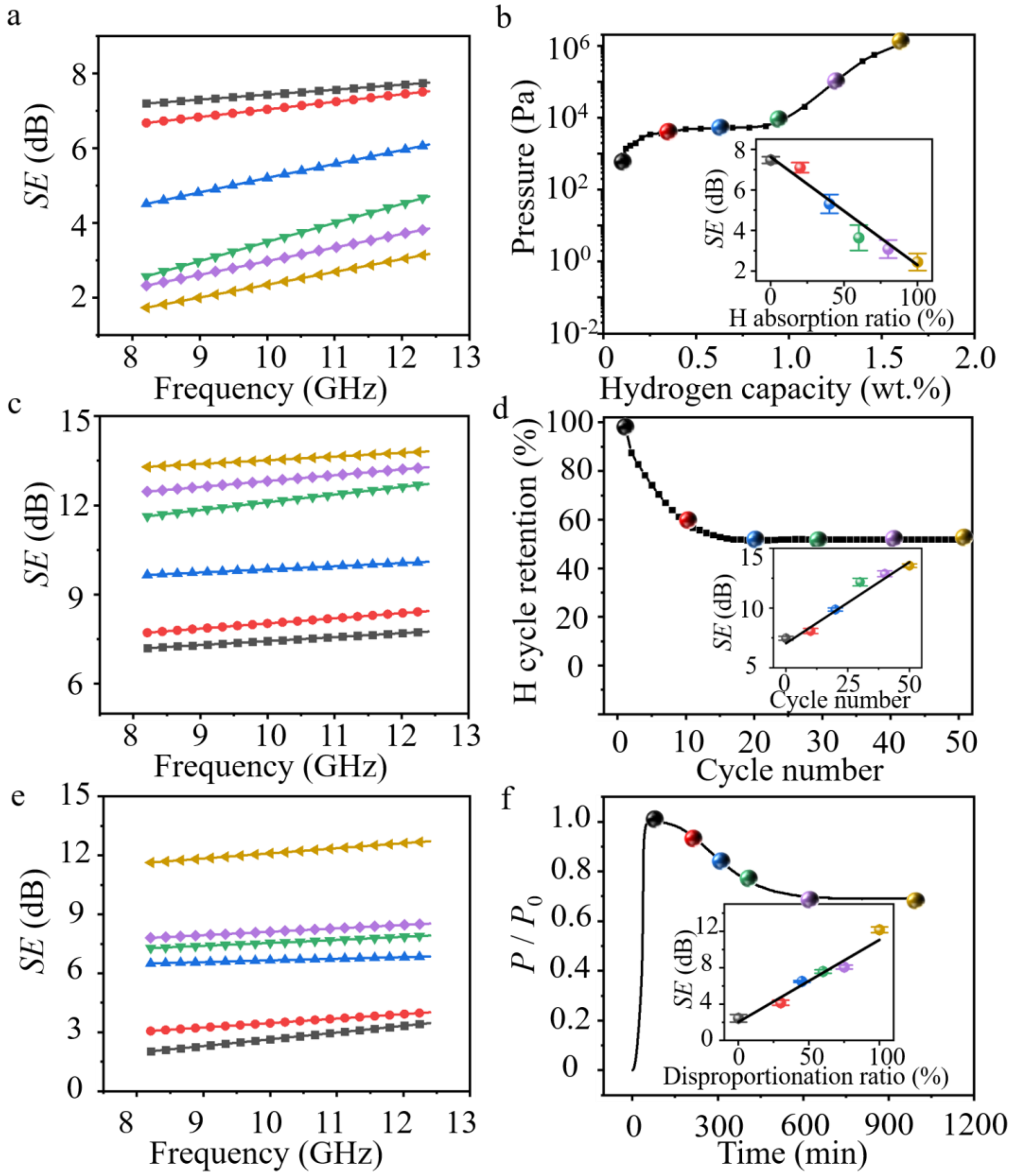

Supplementary Figure 50. Variations of *SE* of ZC-42μm with (**a, b**) hydrogen storage capacity; (**c, d**) cycle number; (**e, f**) disproportionation ratio.

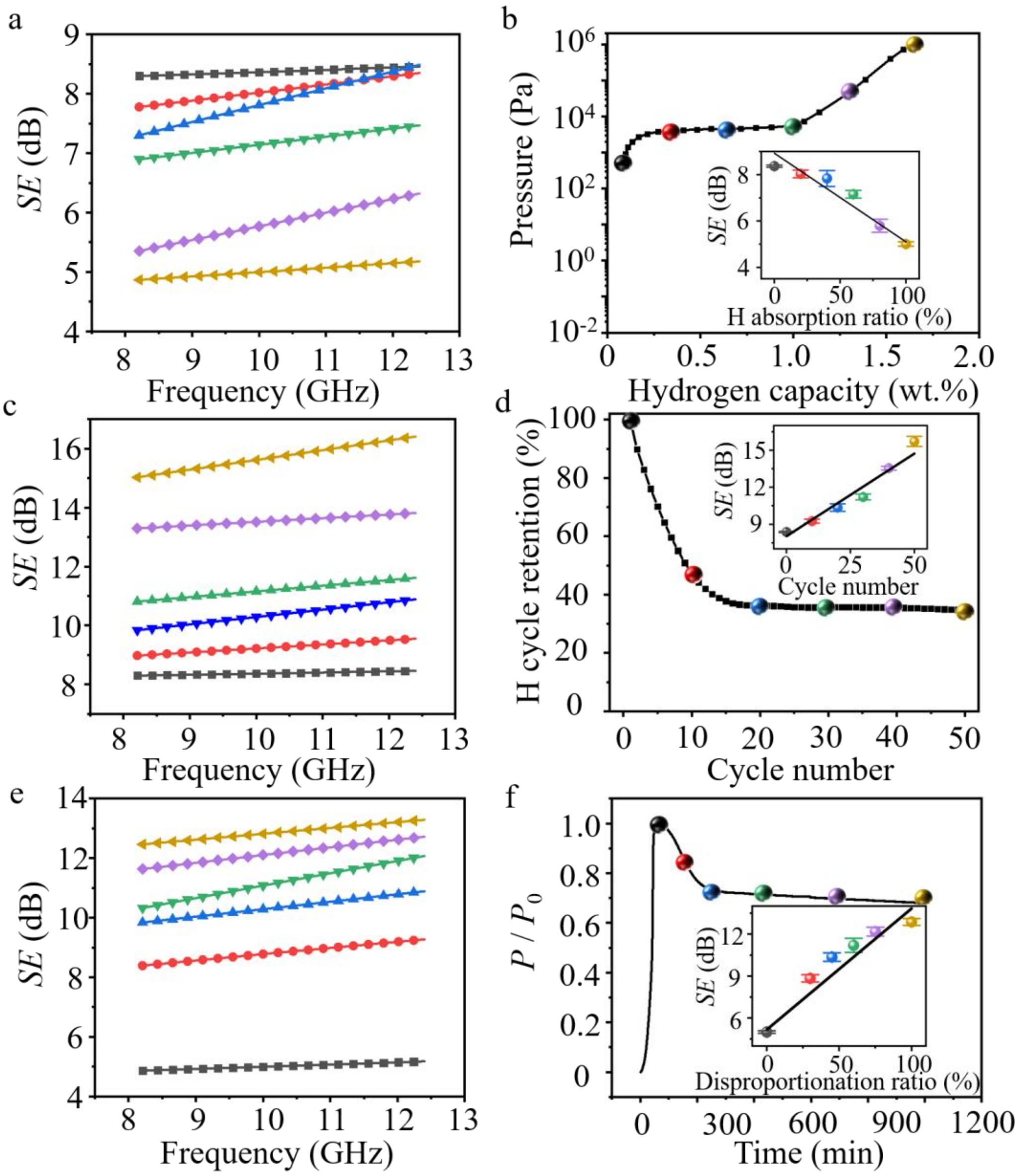

Supplementary Figure 51. Variations of *SE* of ZC-177μm with (**a, b**) hydrogen storage capacity; (**c, d**) cycle number; (**e, f**) disproportionation ratio.

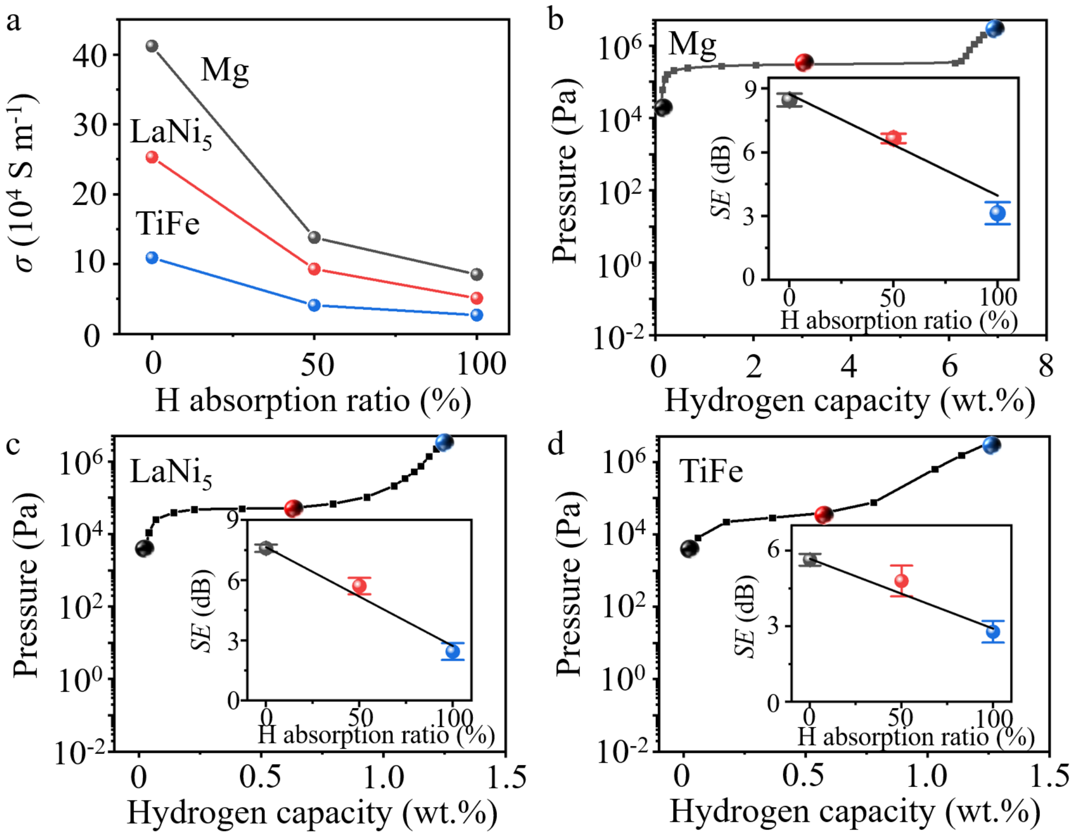

Supplementary Figure 52. (**a**) Variations of electrical conductivity of Mg, $LaNi_5$ and TiFe with H absorption. (**b-d**) Variations of *SE* of Mg, $LaNi_5$ and TiFe with hydrogen storage capacity.

Supplementary References

1. Zhang, X. *et al.* Realizing 6.7 wt% reversible storage of hydrogen at ambient temperature with non-confined ultrafine magnesium hydrides. *Energy Environ. Sci.* **14**, 2302-2313 (2021).
2. Lai, Q. *et al.* How to design hydrogen storage materials? Fundamentals, synthesis, and storage tanks. *Adv. Sustain. Syst.* **3**, 1900043 (2019).
3. Kresse, G. & Furthmüller, J. Efficient iterative schemes for *ab initio* total-energy calculations using a plane-wave basis set. *Phys. Rev. B* **54**, 11169-11186 (1996).
4. Blochl, P. E. Projector augmented-wave method. *Phys. Rev. B: Condens. Matter* **50**, 17953-17979 (1994).
5. Perdew, J. P. *et al.* Atoms, molecules, solids, and surfaces: Applications of the generalized gradient approximation for exchange and correlation. *Phys. Rev. B* **46**, 6671-6687 (1992).
6. Grimme, S. Semiempirical GGA-type density functional constructed with a long-range dispersion correction. *J. Comput. Chem.* **27**, 1787-1799 (2006).
7. Gladys, M. J. *et al.* Comparison of hydrogen and deuterium adsorption on Pd(100). *J Chem. Phys.* **132**, 024714 (2010).
8. Bonev, S. A., Militzer, B. & Galli, G. *Ab initio* simulations of dense liquid deuterium: Comparison with gas-gun shock-wave experiments. *Phys. Rev. B* **69**, 014101 (2004).
9. Chen, H. *et al.* Graphene-based materials toward microwave and terahertz absorbing stealth technologies. *Adv. Optical. Mater.* **7**, 1801318 (2019).
10. Cao, M. *et. al.* Electronic structure and electromagnetic properties for 2D electromagnetic functional materials in gigahertz frequency. *Ann. Phys. (Berlin)* **531**, 1800390 (2019).
11. Wang, S., Cui, Z., Xia, X. & Xue, Y. Size-dependent decomposition temperature of nanoparticles: A theoretical and experimental study. *Phys. B* **454**, 175-178 (2014).
12. Xue, Y., Zhao, M. & Lai, W. Size-dependent phase transition temperatures of dispersed systems. *Phys. B* **408**, 134-139 (2013).
13. Xue, Y., Gao. B., Gao, J. The theory of thermodynamics for chemical reactions in dispersed heterogeneous systems. *J. Colloid Interface Sci.* **191**, 81(1997).
14. Vladimir, M. F. & Edgar, D. Z. Crystal nucleation in silicate glasses: The temperature and size dependence of crystal/liquid surface energy. *J. Non-Cryst. Solids* **265**, 105-112 (2000).
15. Xue, Y. Q., Yang, X. C., Cui, Z. X. & Lai, W. P. The effect of microdroplet size on the surface tension and Tolman length. *J. Phys. Chem. B* **115**, 109-112 (2011).
16. Keene, B. Review of data for the surface tension of pure metals. *Int. Mater. Rev.* **38**, 157 (1993).
17. Chen, H. *et al.* Shape- and size-dependent refractive index sensitivity of gold nanoparticles. *Langmuir* **24**, 5233-5237(2008).
18. Chattaraj, D., Kumar, N., Ghosh, P., Majumder, C. & Dash, S. Adsorption,

dissociation and diffusion of hydrogen on the ZrCo surface and subsurface: A comprehensive study using first principles approach. *Appl. Surf. Sci.* **422**, 394-405 (2017).

19. Chattaraj, D., Parida, S. C., Dash, S. & Majumder, C. Density functional study of vibrational, thermodynamic and elastic properties of ZrCo and $ZrCoX_3$ (X = H, D and T) compounds. *J. Alloys Compd.* **629**, 297-304 (2015).
20. Wang, Q. *et al.* The performance of adsorption, dissociation and diffusion mechanism of hydrogen on the Ti-doped ZrCo(110) surface. *Phys. Chem. Chem. Phys.* **21**, 12597-12605 (2019).
21. Wang, Q. *et al.* Effect of doping Hf on the hydrogen dissociation and diffusion mechanism on the ZrCo (110) surface. *Appl. Surf. Sci.* **483**, 383-390 (2019).
22. Huang, Z., Liu, X., Jiang, L. & Wang, S.Hydrogen storage properties of $Zr_{1-x}Ti_xCo$ intermetallic compound. *Rare Metals* **25**, 200-203 (2006).
23. Kou, H. *et al.* Effects of ball milling on hydrogen sorption properties and microstructure of ZrCo alloy. *Fusion Eng. Des.* **138**, 68-77 (2019).
24. Wan, J. *et al.* Effect of Ni substitution on hydrogen storage properties of $Zr_{0.8}Ti_{0.2}Co_{1-x}Ni_x$ (x = 0, 0.1, 0.2, 0.3) alloys. *Int. J. Hydrogen Energy* **41**, 7408-7418 (2016).
25. Xu, S., Wang, F., Tang, W., Wang, Y. & Yu, R. Microstructure and hydrogen storage properties of $Zr_{0.8}Ti_{0.2}Co_{1-x}Fe_x$ (x = 0, 0.1, 0.2, 0.3) alloys. *Int. J. Hydrogen Energy* **43**, 839-847 (2018).
26. Liang, Z. *et al.* The functioning mechanism of Al valid substitution for Co in improving the cycling performance of Zr-Co-Al based hydrogen isotope storage alloys. *J. Alloys Compd.* **848**, 156618 (2020).
27. Liang, Z. *et al.* A new strategy for remarkably improving anti-disproportionation performance and cycling stabilities of ZrCo-based hydrogen isotope storage alloys by Cu substitution and controlling cutoff desorption pressure. *Int. J. Hydrogen Energy* **44**, 28242-28251 (2019).
28. Yao, Z. *et al.* Improvement on the kinetic and thermodynamic characteristics of $Zr_{1-x}Nb_xCo$ (x = 0-0.2) alloys for hydrogen isotope storage and delivery. *J. Alloys Compd.* **784**, 1062-1070 (2019).
29. Weng, C. *et al.* Effect of Mn substitution for Co on the structural, kinetic, and thermodynamic characteristics of $ZrCo_{1-x}Mn_x$ (x = 0-0.1) alloys for tritium storage. *Int. J. Hydrogen Energy* **42**, 28498-28506 (2017).
30. Zhang, G. *et al.* Effects and mechanism of Ti, Ni, Sc, Fe substitution on the thermal stability of zirconium cobalt-hydrogen system. *Int. J. Hydrogen Energy* **40**, 6582-6593 (2015).
31. Peng, L., Jiang, C., Xu, Q. & Wu, X. Hydrogen-induced disproportionation characteristics of $Zr_{1-x}Hf_xCo$ (x = 0, 0.1, 0.2 and 0.3) alloys. *Fusion Eng. Des.* **88**, 299-303 (2013).
32. Jat, R. A. *et al.* An analogy of interstitial site occupancy and hydrogen induced disproportionation of $Zr_{1-x}Ti_xCo$ ternary alloys. *Int. J. Hydrogen Energy* **42**, 8089-8097 (2017).
33. Liang Z. *et al.* Positive impacts of tuning lattice on cyclic performance in ZrCo-

based hydrogen isotope storage alloys. *Mater. Today Energy* **20**, 100645 (2021).
34. Jat, R. A. *et al.* Structural and hydrogen isotope storage properties of Zr-Co-Fe alloy. *Int. J. Hydrogen Energy* **40**, 5135-5143 (2015).
35. Yao, Z. *et al.* An impact of hydrogenation phase transformation mechanism on the cyclic stabilizing behavior of $Zr_{0.8}Ti_{0.2}Co$ alloy for hydrogen isotope handling. *Mater. Today Energy* **18**, 100554 (2020).
36. Jat, R. A., Parida, S. C., Agarwal, R. & Kulkarni, S. G. Effect of Ni content on the hydrogen storage behavior of $ZrCo_{1-x}Ni_x$ alloys. *Int. J. Hydrogen Energy* **38**, 1490-1500 (2013).
37. Zhao, Y. *et al.* Effect of Ti substitution on hydrogen storage properties of $Zr_{1-x}Ti_xCo$ ($x = 0, 0.1, 0.2, 0.3$) alloys. *J. Energy Chem.* **23**, 9-14 (2014).
38. Yao, Z. *et al*. Achieving excellent cycle stability in Zr-Nb-Co-Ni based hydrogen isotope storage alloys by controllable phase transformation reaction. *Renew. Energy* **187**, 500-507 (2022).
39. Qi, J. *et al*. Effect of isostructural phase transition on cycling stability of ZrCo-based alloys for hydrogen isotopes storage. *Chem. Eng. J.* **455**, 140571 (2023).
40. Shahzad, F. *et al.* Electromagnetic interference shielding with 2D transition metal carbides (MXenes). *Science* **353**, 1137-1140 (2016).
41. Iqbal, A. *et al.* Anomalous absorption of electromagnetic waves by 2D transition metal carbonitride Ti_3CNT_x (MXene). *Science* **369**, 446-450 (2020).
42. Li, Y. *et al.* Multifunctional organic-inorganic hybrid aerogel for self-cleaning, heat-insulating, and highly efficient microwave absorbing material. *Adv. Funct. Mater.* **29**, 1807624 (2019).
43. Zhang, M. *et al.* Electromagnetic absorber converting radiation for multifunction. *Mater. Sci. Eng. R Rep.* **145**, 100627 (2021).
44. Gao, Z., Lan, D., Zhang, L. & Wu, H. Simultaneous manipulation of interfacial and defects polarization toward Zn/Co phase and ion hybrids for electromagnetic wave absorption. *Adv. Funct. Mater.* **31**, 2106677 (2021).
45. Wu, Z. *et al.* Dimensional design and core-shell engineering of nanomaterials for electromagnetic wave absorption. *Adv. Mater.* **34**, 2107538 (2022).
46. Cao, M. *et al.* Variable-temperature electron transport and dipole polarization turning flexible multifunctional microsensor beyond electrical and optical *energy. Adv. Mater.* **32**, 1907156 (2020).
